# Sustainable char production from pyrolysis of refuse-derived fuel for nitrate removal from water

**DOI:** 10.1038/s41598-026-51297-1

**Published:** 2026-05-21

**Authors:** Yashasvi Trivedi, Abhishek Sharma, Manisha Sharma, Ranjeet Kumar Mishra, Jyeshtharaj B. Joshi

**Affiliations:** 1 Department of Biotechnology and Chemical Engineering, Manipal University Jaipur, Jaipur, Rajasthan 303007 India; 2 Manipal Institute of Technology, Manipal Academy of Higher Education, Manipal, India; 3 Department of Chemical Engineering, Institute of Chemical Technology, Mumbai, Maharashtra 400019 India; 4 Department of Chemical Engineering, BITS Pilani K. K. Birla Goa Campus, Zuarinagar, Sancoale, Goa 403726 India

**Keywords:** RDF char, Fixed-bed column, Pyrolysis, Adsorption kinetics, Nitrate removal, Waste valorisation, Chemistry, Engineering, Environmental sciences

## Abstract

This study explores a sustainable approach to simultaneously valorising municipal solid waste and remediating nitrate-contaminated water by generating an engineered adsorbent from refuse-derived fuel (RDF) via a thermochemical pathway. RDF feedstock was pyrolysed at 500 °C in a pilot-scale rotary kiln to produce RDF500 char, subsequently modified using zinc chloride in 2:1 wt.% ratio (RDF500-2) to enhance surface functionality and adsorption performance. Modification with ZnCl_2_ has been extensively studied, and when handled properly, it does not pose greater environmental risks, thereby supporting sustainability aspects. Furthermore, characterisation through FTIR, BET, and SEM confirmed the introduction of oxygenated groups (–OH, –COOH, –C=O), increased surface heterogeneity, and the development of a micro-mesoporous structure conducive to nitrate adsorption. Fixed-bed column experiments were performed under varying operational conditions, including bed height (5–10 cm), influent flow rate (3–7 mL min^-1^), and initial nitrate concentration (67–185 mg L^-1^) utilising RDF500-2, to determine breakthrough behaviour and adsorption kinetics. The maximum adsorption capacity was 21.32 mg g^-1^, corresponding to 89% nitrate removal efficiency under optimal conditions. The kinetic modelling using Thomas, Yoon–Nelson, Adams–Bohart, Wolborska, and Dose–Response models exhibited an excellent correlation (R^2^ > 0.98), with the Dose–Response and Thomas models showing the best predictive accuracy for column optimisation. A scale-up analysis for a 100 L min^-1^ continuous flow system predicted 80% removal efficiency, confirming the practical applicability of modified RDF char (RDF500-2).

**Supplementary Information:**

The online version contains supplementary material available at https://doi.org/10.1038/s41598-026-51297-1.

## Introduction

The current state of environmental pollution and improper solid waste management is already alarming. Rapid urbanisation, population growth, inadequate disposal practices, open dumping, landfill deposition, and inefficient treatment of municipal solid waste (MSW) have led to a significant increase in waste generation, severely impacting environmental quality, public health, and economic sustainability. Among these challenges, the uncontrolled release of solid waste and liquid pollutants into water bodies remains one of the most critical sources of environmental degradation^1^. Nitrate contamination levels in water bodies have surpassed the permissible limits (45 mg/L NO_3_^-^N as prescribed by WHO) in several countries, including India, Australia, the United Kingdom, Saudi Arabia, China, Iran, and North America. In India, the highest reported nitrate contamination in groundwater has been approximately 2000 mg/L, substantially exceeding regulatory standards^2^. Moreover, prolonged nitrate-contaminated water consumption causes cyanosis, methemoglobinemia (blue baby syndrome) in infants, thyroid dysfunction, gastrointestinal distress and central nervous system-related disorders in humans^3^. Pollution measures and water quality were analysed using the new approach, the “Water pollution index (WPI),” which indicated a value of around 1.94 for Indian groundwater, placing it in the highly polluted water category^4^. Various techniques, including oxidation, adsorption, reverse osmosis, evaporation, and ion-exchange, have been employed to eliminate a wide range of wastewater pollutants^5^. The most efficient, successful, and feasible method for removing numerous compounds is adsorption^6^^7^. Solid waste (SW) comprises leftovers from commercial and household activities, along with biodegradable compounds, food waste, paper, wood, plastics and microplastics, fibres, textiles from industries, electrical and hazardous waste materials, as well as biodegradable, toxic, and inert waste^8^. According to the Global Waste Management (Waste 2.0), the amount of solid waste generated each year is 2.01 billion tonnes, and by 2050, it is expected to reach 3.8 billion tonnes^9^. Both commercial and residential sources contribute to MSW. MSW was estimated to be 55–65% from residential sources, including waste from several family units and houses. Commercial waste sources account for 35–45% of MSW generation, including waste from schools and some industrial sites where packaging is produced^10^. Municipal waste has been a significant global issue, particularly in developing nations, where it is often dumped openly or deposited in landfills. Composting, landfilling, recycling and waste-to-energy are four basic elements of MSW industries^11^. Over 62 million tons of solid waste are produced in India annually, according to a survey by The Energy and Resources Institute (TERI)^12^. Converting MSW into energy (through incineration and gasification) faces various scaling-up challenges^13,14^. However, pyrolysis at mild conditions provides the opportunity to produce secondary raw materials, such as char, oil, and syngas^15^. MSW after mechanical biological treatment (MBT) produces the highly combustible fraction, RDF, which, upon pyrolysis, yields high-calorific syngas and consistent char quality. RDF primarily consists of wastepaper (3–9%), plastics (19–35%), textiles (2–9%), yard waste (20%), and biomass (10–20%), including kitchen and other waste (10–12%)^16–20^. RDF contains lower levels of pollutants (such as cadmium and chlorine) and higher levels of combustibles than MSW^21^. Combustible shredded bulky waste is sent to RDF facilities via municipal solid waste incinerators^22^. Thermal behaviour and kinetic parameters are significantly affected by interactions among the various components of RDF. India has the potential to produce 12 million tons of RDF char from solid waste^23^. 40% of the total MSW is converted into the RDF, and 30–40% of RDF on pyrolysis is converted into char, which is available in India and can be utilised as secondary raw materials and for the application in composite, filler materials and nanomaterials as an adsorbent for water treatment through the adsorption process^24^. In another study, MSW-Biochar was utilised as an adsorbent for toluene and m-xylene adsorption and further evaluated as a source of volatile organic compounds (VOCs) and their chemical properties^25^. Another study found that municipal solid waste-generated chars exhibited promise as adsorbents (produced through gasification) for wastewater treatment^11^. The study reported that heavy metals may be remediated using sustainable solid waste biochar in landfill leachate^26^. Newly formulated adsorbents derived from municipal solid waste and pure clay mineral (montmorillonite) were utilised for the removal of ionised adsorbates^1^. Another study used a bench-scale column investigation with walnut shell and cotton gin prepared biochar to remove pharmaceuticals from water^27^. This study explored the significance of textile industrial sludge (TIS)—prepared biochar in removing ofloxacin (a pharmaceutical compound) from a water-based solution^28^. A study demonstrated the sorption of sulfamethoxazole onto polyethene microplastic waste in an aquatic environment^29^. The present study addresses the existing research gap in the sustainable management of municipal solid waste (MSW) and the simultaneous treatment of nitrate-contaminated water, two of the most pressing environmental challenges globally. Although extensive research has been conducted on biochar and activated carbon for adsorption-based pollutant removal, very limited attention has been given to utilising RDF as a precursor to generate engineered adsorbents via controlled thermochemical pathways. Previous studies have largely focused on small-scale batch adsorption using biomass or agricultural residues; however, the lack of pilot-scale synthesis, systematic column optimisation, and model-based interpretation for RDF-derived adsorbents remains a critical research gap. Furthermore, no comprehensive study has examined the column-scale nitrate adsorption performance of chemically modified (zinc chloride) RDF char (RDF500-2) with statistical modelling and scale-up analysis. The novelty of this work lies in developing a pilot-scale rotary kiln system to produce RDF500-2 and evaluating its performance for continuous nitrate removal in a fixed-bed column under varying operational conditions (bed height, initial nitrate concentration, and influent flow rate). The study integrates five kinetic and empirical models, Thomas,Adams–Bohart, Wolborska, Yoon–Nelson, and Dose–Response, to identify the most reliable predictive model for column design and optimisation. This research uniquely combines waste valorisation with water purification by demonstrating a “waste-to-treat-waste” approach, highlighting modified RDF char as a low-cost, scalable, and sustainable alternative to commercial adsorbents. The proposed system provides novel insights into kinetic modelling, process optimisation, and the real-world applicability of integrated waste management.

Compared to mixed MSW chars, RDF char provides a more consistent feedstock, which varies significantly in composition and adsorption behaviour. ZnCl_2_ activation is known to add Lewis-acidic and oxygenated groups that bind nitrates more efficiently than adsorption by surface area alone. To the best of our knowledge, no research has been conducted on the applicability of efficient modified RDF char as an adsorbent in a column for nitrate removal from wastewater. The present study focuses on evaluating the adsorption performance and scale-up feasibility using pilot-scale design parameters (EBCT, bed height, and adsorbent mass requirements). Therefore, the study’s objective was to investigate groundwater nitrate removal utilising a fixed-bed column containing pyrolysed (at 500 °C) engineered MSW-derived RDF char (RDF feedstock was procured from BEIL, Bharuch, Gujarat, India). RDF char has been considered a novel, promising, and sustainable waste-derived low-cost adsorbent, due to its scalability, heterogeneity, practicality, real waste valorisation, and diverse functionality (–OH, –COOH, –C=O) features, as well as its alignment with circular economy principles and integration of waste management strategies and environmental policy applications globally. This research introduces a novel waste-to-treat-waste framework that strongly supports SDG 6 (clean water and sanitation) by enabling efficient wastewater remediation using waste-derived materials. The approach promotes SDG 11 (sustainable cities and communities) through sustainable waste management and resource recovery, while advancing SDG 12 (responsible consumption and production) by valorising waste within a circular economy framework. Furthermore, converting waste into functional treatment materials contributes to SDG 13 (climate action) by reducing landfill disposal, lowering carbon emissions, and supporting low-carbon environmental remediation strategies.

## Materials and methods

### RDF char production

Solid waste generated was used to produce RDF feedstock in the laboratory (waste to resources) of Manipal University, Jaipur, utilising a pilot-scale rotary kiln reactor. RDF feed contained (weight %, avg. values of triplicate sorting), paper (3.95), yard waste (19.54), wood (5.21), clothes (7.65), rubber (3.01), plastics (38.80), mixed waste- inorganics, dirt, threads, and others (21.9) by manual sorting from a large batch received from BEIL MBT plant in Coimbatore, India. RDF was washed and dried overnight prior to pyrolysis. The RDF was pyrolysed at 500 °C (RDF500) to achieve better adsorption performance^30,31^ at a feed rate of 2 kg/h, with preheating at 150 °C for moisture removal. Condensable gaseous products and air in the zone of pyrolysis were eliminated through pure nitrogen. Prior to procurement, TGA analysis was performed to evaluate the thermal behaviour of the RDF sample under a pure nitrogen atmosphere (100 mL/min) while heating from 20 °C to 900 °C at a rotary speed of 1 revolution per minute.

After the completion of the pyrolysis process and natural cooling of the interior of the rotary kiln to room temperature, the char was accumulated from the rotary drum. To prevent the accumulation of residual tar, ash, or fine particulates in the gas-handling lines, the system was cleaned after each run. This included manual removal of remaining solid deposits from the drum walls, followed by flushing the gas outlet lines with nitrogen to eliminate the condensed vapours and particulates. Any detachable sections, such as condensers, filters, and connecting pipes, were rinsed and dried. This routine cleaning ensured consistent operating conditions and prevented contamination or blockages in subsequent experiments. After sieving and reducing the collected material to particles between 1.5 and 2.5 mm, the material was chemically modified with ZnCl_2_. Modification of the adsorbent plays a crucial role in improving the adsorption process by enhancing oxygen-containing surface functional groups. This leads to the formation of inner-sphere and electrostatic binding sites, which influence the surface chemistry. Consequently, the adsorption capacity of pristine char for the targeted pollutant is enhanced.

### Modification of RDF char

The procured RDF char undergoes the ash removal treatment by NaOH washing of the char by a protocol stated^32^. RDF char (20 g) was treated for 24 h at 250 rpm and at 35 °C with 250 mL of 1N NaOH. Furthermore, the chemical activation of unmodified RDF char (RDF500) with zinc chloride enhanced the characteristics of the RDF char. For this purpose, the weight proportion of 2:1 of ZnCl_2_: biochar was optimised from batch studies highlighted in one of our research articles^33^ among three wt. ratios (1:1, 2:1, 3:1). The char was calcined at 700 °C in an inert atmosphere (presence of N_2_ gas) in muffle furnace. The char was rinsed several times with 0.05 M HCl, then with distilled water, until the pH reached neutrality to remove inorganics. Upon modification, the sample was further labelled as RDF500-2 (2:1). Based on the results from the batch adsorption experiments, RDF500-2 was employed in the fixed-bed column studies (as shown in Fig. S1) conducted in the present work. Zinc chloride (ZnCl_2_) concentrated 35% pure hydrochloric acid (HCl), and potassium nitrate (KNO_3_) (97% pure) were purchased from Loba Chemie Pvt Ltd. and used as supplied. Analytical-grade chemical reagents were utilised in this study.

### Feed solution (adsorbate) preparation

Initially, a KNO_3_ solution of 100–350 mg/L was prepared using de-ionised (DI) water in ambient conditions. Subsequent dilutions were made to obtain working solutions (simulated water) of varying nitrate concentrations (68 to 190 mg/L). Nitrate uptake was monitored spectrophotometrically using a UV–vis double-beam spectrophotometer (UV1900i, Shimadzu) with a range of characteristic wavelengths (207–209 nm) at varying concentrations. The performance of RDF char was evaluated and compared based on its nitrate adsorption uptake capacity (mg/g) and removal potential. To minimise error and to ensure reproducibility, every experiment was carried out in duplicates.

### Characterisation of RDF feedstock and char

The thermogravimetric analysis (TGA) for both RDF feedstock and char procured from a similar source, on which work^21^ has been carried out by our group at Manipal University, Jaipur. The ultimate and proximate analyses of RDF feedstock and char are highlighted in Section "Results and discussions". Sophisticated instruments (FTIR spectroscopy, BET analysis, FESEM and EDS) were employed to investigate the surface characteristics of modified and unmodified adsorbents. Surface functional groups of RDF500, RDF500-2 and spent RDF500-2 were identified using Fourier Transform Infrared (FTIR) spectroscopy, which was carried out using the KBr pellet method on a Bruker ALPHA II spectrometer.

The FTIR spectrometer is equipped with an attenuated total reflectance (ATR) model, featuring a standard ZnSe beam splitter, suitable for solid-to-liquid samples within an operating pH range of 4–8. The instrument offers a signal-to-noise ratio of 50,000:1 (peak-to-peak) with a spectral resolution of 0.8 cm^-1^. Surface morphology was determined by Scanning electron microscopy (SEM) (make: JEOL Model: JSM-7610F). The operating system operates within a distance range of 6–40 mm and at 30 kV for SEM analysis. At vacuum conditions ranging from 10^–4^ to 0.9 kPa, nitrogen was used as the imaging gas, with magnification ranging from 10^–4^ to 10^6^ times, and it has been found suitable for imaging high-vacuum, dry, non-conductive substances. The Brunauer–Emmett–Teller (BET) analysis was used to quantify the pore volume, average particle size, specific surface area and pore size distribution using a 4LX analyzer QUANTACHROME NOVA touch. For degasification, the samples (approximately 100–150 mg) were placed in ports to allow nitrogen gas/helium to flow from 1 to 100 kPa, after which the system was subjected to a vacuum condition. Furthermore, the gases are expected to flow at 180 °C for 6 h. Once the temperature reaches the set value, a holding time of 1–2 h is provided. Also, the degassing pressure is maintained at 0 bar for zero air and 1 bar for nitrogen, respectively. The samples are then allowed to cool in the ports for 15 min. The samples were nitrogen-adsorbed at 77 K under ambient pressure after being degassed for 8–10 h at 180–200 °C under vacuum. Finally, the results were analysed using BEL MASTER software, integrated with the system and employing the BJH model to determine pore size distribution. Additionally, DFT (density functional theory) analysis, nitrogen adsorption–desorption isotherms, and BET plots were used for comprehensive characterisation of the pore structure. A JEOL JSM-7610F microscope was used for Field Emission Scanning Electron Microscopy (FESEM) to study the surface morphology of all char materials at different resolutions (× 1000, × 3000).

### Fixed-bed column experiments

**Fig. 1:**
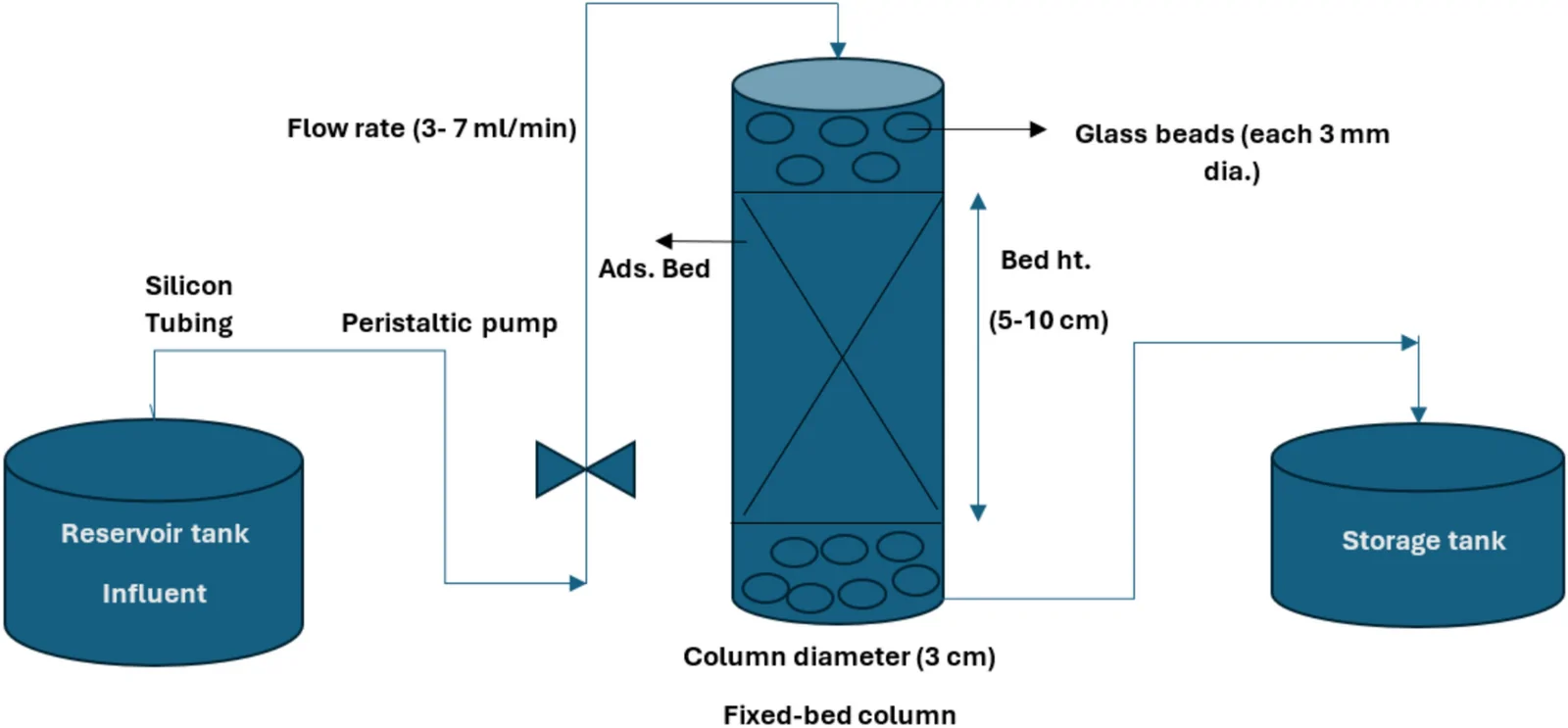
Diagrammatic representation of a continuous fixed-bed column for nitrate adsorption on RDF500-2.

Removal of nitrate was investigated for RDF500-2 through the fixed-bed column studies. The arrangement consisted of a vertical glass adsorption column with an inside diameter of 3 cm and a total length of 70 cm, as shown in Fig. 1. The column was filled with RDF500-2 char, with varying adsorbent bed heights ranging from 5 to 10 cm and contained 6–20 g of adsorbent. The nitrate stock solution (KNO_3_ simulated water) with a concentration varying from 100 to 300 mg/L (NO_3_^-^ concentration: 65–190 mg/L) was circulated by a peristaltic pump at flow rates of 3 to 7 mL/min from an influent reservoir (10 L at 300 mL/min). Dry packing was preferred over wet packing to prevent the suspension of char particles. The first 3 cm of the thick bottom layer of the column was filled with glass wool, then the second column layer of 2 cm was filled with the 3 mm-diameter spherical glass beads to provide uniform flow distribution across the column’s cross-section. The third bottom layer of the column was fixed with the circular mesh screen (18 mesh size) to avoid the deposition of the char particles in the voids of glass beads, and finally, the bed of adsorbent (RDF500-2) was added as the topmost layer of the continuous column, shown in Fig. S2. Distilled water was flushed into the system (column) 2 to 3 h prior to the nitrate (adsorbate) flow. The samples were taken at regular intervals from a storage tank connected to the column’s exit. The experiment was performed for 6 to 7 h, depending on the exhaustion time or until equilibrium was achieved, in different studies that varied the adsorbent bed height, initial adsorbate concentration, and adsorbate flow rate, respectively. Samples were collected between 10 and 15 min. Furthermore, all experiments were conducted in duplicate, and the average values were reported. The breakthrough and exhaustion points of the columns were determined using the BTCs. The breakthrough occurs when the effluent concentration reaches 5% of the influent (initial adsorbate concentration) or when the ratio of the influent and effluent concentrations reaches 5% (C_t_/C_0_ = 0.05). However, in the current study, a breakthrough is expected to occur when the influent concentration reaches 40 ppm, corresponding to the selected threshold of the permissible nitrate limit of 45 ppm of nitrate nitrogen (NO3-N) for breakthrough analysis. The exhaustion point was defined as Ct/C_0_ = 0.95, at which the effluent concentration reached 95% of the influent concentration. The pH (6.9) and the temperature (35 °C) were optimised in one of our batch studies^33^, utilising the RDF char as a nitrate removal adsorbent, and therefore the effect of flow rate, bed height and initial nitrate concentration on uptake capacity and removal efficiencies were studied in the present work of fixed bed column adsorption. The hydrodynamic coefficient influences mass transfer factors and adsorption equilibrium, as well as operational parameters, including bed height (Z, cm), inlet concentration (C0, mg/L), and feed flow rate (Q, mL/min), which, in turn, determine the BTC shape^34^. BTCs were analysed based on the parameters: time of breakthrough (t_b_), exhaustion time (t_s_), breakthrough adsorption uptake capacity (q_b_), uptake capacity at saturation (q_s_), removal efficiency (Y, %) at saturation, sorbent usage rate (SUR) and degree of sorbent used (DoSU). These are obtained as follows:

The quantity of nitrate fed in the column (m_in, t_, mg) to time, t is

1 $${m}_{in, t }={C}_{0}Qt$$

where Q is flow rate (L/h), C_0_ is initial nitrate concentration (mg/L),

The amount of nitrate exiting the column or the quantity of nitrate unabsorbed (m _out, t,_ mg) can be defined as the area under the BTC ((dimensionless) multiplied by flow rate, Q (mL/min) and inlet concentration (C_0_, mg/L) up to time t^34^.

2 $${m}_{out, t }={C}_{0}Q {\int}_{0}^{t}\frac{{C}_{t}}{{C}_{0}}$$

The amount of nitrate adsorbed in the column at time t (m _ad, t_, mg) was calculated as:

3 $${m}_{ ad,t }={m}_{in, t }- {m}_{out, t}$$

The equilibrium adsorption capacity (q_e_, mg/g) up to saturation can be determined by dividing the mass of nitrate adsorbed in total at saturation (m _ad, t_, mg) by the mass of adsorbent (nitrate) packed in the column (M, g):

4 $${q}_{e}=\frac{{m}_{ ad,{t}_{s}}}{M}= \frac{{C}_{0}Q {\int}_{0}^{t}\left(1-\frac{{C}_{t}}{{C}_{0}}\right)dt}{M}$$

Likewise, the breakthrough uptake capacity can also be determined as:

5 $${q}_{b}=\frac{{m}_{ ad,{t}_{b}}}{M}= \frac{{C}_{0}Q {\int}_{0}^{{t}_{b}}\left(1-\frac{{C}_{t}}{{C}_{0}}\right)dt}{M}$$

Another parameter, SUR (g/L), is defined as the mass of adsorbent required to treat 1 unit volume of influent solution until breakthrough occurs. It quantifies how fast the adsorbent is used relative to the treated solution volume:

6 $$SUR=\frac{M}{{V}_{b}}=\frac{M}{Q{t}_{b}}$$

where V_b,_ L is the breakthrough volume.

At saturation, removal efficiency (Y) is calculated as the proportion of total nitrate fed into the column (m_in, ts_, mg) that has been adsorbed (m _ad, t_, mg).

7 $$Y= \frac{{m}_{ ad,{t}_{s}}}{{m }_{in, {t}_{s} }}=\frac{{m}_{ ad,{t}_{s}}}{{C}_{0}Q{t}_{s}}$$

DoSU is defined as the ratio of the adsorption capacity at breakthrough (q_b_, mg/g) to the capacity at equilibrium (q_e_, mg/g):

8 $$DoSU=\frac{{q}_{b}}{{q}_{e}}$$

DoSU indicates how efficiently the adsorbent material is utilised before reaching saturation. When DoSU equals 1, it signifies 100% utilisation of the bed’s maximum potential.

EBCT is equal to the bed volume divided by the adsorbate feed flow rate, calculated in minutes. It is determined as:

9 $$EBCT=\frac{Bed volume}{Flow rate}$$

where the bed volume defines the volume of the adsorbent bed, which is calculated in mL, and the influent flow rate is calculated in mL/min.

### Statistical methods

Numerous basic empirical models were used to interpret the compelling behaviour and to evaluate certain coefficients. Determining the design conditions for the column is the goal of modelling the breakthrough curve. To accurately capture the effects of film diffusion resistance, axial dispersion, and intra-particle diffusion (including both pore and surface diffusion), a considerable number of mass transfer criteria must be considered. In this study, the following mathematical models were used to determine the best fit for the experimental data. The model fitting was performed by first calculating Ct/C_0_ from experimental data and selecting the appropriate range (for Wolborska model, Ct/C_0_ < 0.1–0.2). The required linearized form of the model (e.g., ln (Ct/C_0_) vs. time) was then plotted (origin/excel). A linear trendline was applied, and model parameters were obtained from the slope and intercept. The goodness of fit was evaluated using R2 from the trendline, while RMSE was calculated separately using predicted and experimental values.

#### Thomas model

Assuming pseudo-second order adsorption kinetics, this model is based on the Langmuir adsorption isotherm. The following is the model’s expression^35^:

10 $$\frac{{C}_{t}}{{C}_{0}}=\frac{1}{1+\mathrm{exp}[\left(\frac{{k}_{Th}{q}_{Th}M}{Q}-{k}_{Th}{C}_{0}t\right)]}$$

where, k_Th_ is Thomas rate constant (L h^-1^ mg^-1^) and q _Th,_ mg/g is the equilibrium adsorption capacity.

#### Yoon Nelson model

The Yoon–Nelson model predicts that each adsorbate molecule has a probability of adsorption that decreases with the likelihood of breakthrough and adsorption. The model is limited by assuming symmetrical breakthrough curves, even though the actual system may not always behave symmetrically. Also, accounts for less accuracy at high mass transfer resistances and for multi-component adsorption. The Yoon and Nelson (YN) model expression was calculated^34^:

11 $$\mathrm{ln}\frac{{C}_{t}}{{C}_{0}-{C}_{t}}= {k}_{YN}- {\tau k}_{YN}$$

where Yoon–Nelson rate constant (min^-1^) is given as kYN, which determines how fast breakthrough occurs and the time when 50% of the column is exhausted (when half of the adsorbate concentration appears in the effluent) is given by τ.

#### Adams–Bohart model

The model states that the adsorption rate is determined by the adsorbate concentration and the adsorbent’s residual capacity. The surface reaction theory predicts irreversible adsorption, ideal plug flow, and an equilibrium that is not intermediate, and serves as the basis for the development of the model. The simplified form of the model is given as^36^:

12 $$\frac{{C}_{t}}{{C}_{0}}=\frac{1}{1+\mathrm{exp}[\left(\frac{{k}_{BA}{N}_{O}Z}{u}-{k}_{BA}{C}_{0}t\right)]}$$

where Bohart–Adams adsorption rate constant (L/mg min) is given by k_BA_, dynamic bed adsorption capacity (mg/L) is expressed as N_0_, bed height (Z, cm), t is saturation (total) time (min), and linear flow rate (cm/min) is given by u.

#### Dose–response model

Dose–response model predicts that as the exposure time increases, the adsorbent gradually becomes saturated, and the fraction of breakthrough increases according to a sigmoidal breakthrough curve. Interpreting the model in more depth, the “dose” represents the exposure time to the adsorbent, and the “response” quantifies the adsorbate that remained unabsorbed. All the above models predicted a value (non-zero) of Ct/Co at t = 0, which deviates from the real adsorption dynamics. Therefore, a dose–response model was employed to formulate breakthrough curves, thereby overcoming this shortcoming^34^:

13 $$\frac{{C}_{t}}{{C}_{0}}=1-\frac{1}{1+{\left(\frac{t}{\beta }\right)}^{\alpha }}$$

The time at which the effluent concentration reaches 50% of the influent concentration is inferred by β, whereas α is the dose–response constant.

#### Wolborska model

The breakthrough curves were primarily described at low concentrations using Wolborska model. It is predicated that at high solution flow rates, there is little or no axial dispersion. The Wolborska model’s linear form is shown as^37^:

14 $$\mathrm{ln}\left(\frac{{C}_{t}}{{C}_{0}}\right)= \frac{{\beta}_{a}{C}_{0}t}{{N}_{O}}- \frac{{\beta}_{a}Z}{u}$$

The slope and intercept of the model can be interpreted by a linear plot of ln (C_t_/C_0_) and t from the model constant N_0_ and β_a,_ respectively, and β_a_ is the external mass transfer kinetic coefficient (min^-1^).

#### Analysis of sample

To ensure experimental rigour, reagent blanks and standard solutions were included with the column. Satisfactory results from standard solutions (97–98%) verified that all glassware was free from contamination. All column experiments were carried out twice, and the average results are documented in the findings. Effluent samples (5 mL) were initially collected in 15 mL glass test tubes. The samples were then filtered through 0.45 µm syringe filters after withdrawing at regular time intervals (10 and 15 min) for various column-based experimental studies. To analyse the concentration of nitrate through absorbance in a UV-spectrophotometer (Shimadzu UV1900i), dilute HCl of 0.1 mL was added to the effluent sample (KNO_3_ simulated water) of 5 mL^38^. Furthermore, the samples were analysed at their characteristic wavelengths of 208–210 nm (which varied with flow rate, initial adsorbate concentration, and bed height), prior to which calibration standards were prepared.

## Results and discussions

### Characterisation of feedstock

The physicochemical characterisation of the RDF feedstock and char produced at 500 °C by thermal conversion demonstrates significant transformations (highlighted in Table 1**)**. The proximate analysis shows that the raw RDF contains high volatile matter (60.5 wt.%) and moderate moisture (10.2 wt.%), reflecting its heterogeneous and partially biodegradable nature. The moisture content is drastically reduced to 1.80 wt.%, indicating effective dehydration and improved fuel stability^39^. A decrease in volatile matter to 11.20 wt.% is observed, accompanied by a significant increase in fixed carbon from 12.80 to 52.40 wt.%. This shift confirms extensive devolatilization and carbon enrichment during char formation. The ash content increases from 16.50 wt.% in the feedstock to 34.6 wt.% in the char, primarily due to the concentration of inorganic minerals after the removal of organic volatiles, a common characteristic of thermochemically treated waste-derived materials^40^. The ultimate analysis further corroborates these trends. The carbon content increases from 50.4 wt.% in the RDF feed to 58.2 wt.% in the char, highlighting enhanced carbonisation and the formation of a more aromatic and condensed carbon structure^41^. In contrast, hydrogen content decreases sharply from 6.20 wt.% to 1.30 wt.%, while oxygen content reduces from 32.1 wt.% to 20.8 wt.%, resulting in dehydration and decarboxylation reactions during pyrolysis^42^. These changes indicate increases in the C/H and C/O ratios, indicative of improved thermal stability of the char. A slight increase in nitrogen content (from 1.10 wt.% to 1.50 wt.%) is attributed to its relative enrichment as other elements are volatilized, whereas sulphur shows a marginal increase from 0.22 wt.% to 0.28 wt.%. Importantly, the chlorine content decreases from 0.75 wt.% to 0.52 wt.%, which is beneficial from an environmental and corrosion concern, as chlorine is associated with HCl emissions and fouling during combustion. These changes are reflected in the higher heating value (HHV), which increases from 20.10 MJ/kg for the RDF feedstock to 21.8 MJ/kg for the char^43^. Despite the increased ash content, the higher HHV indicates improved energy density due to rich carbon content and reduced elements (O, S, N and Cl atoms). The RDF char produced at 500 °C exhibits characteristics of a more carbon-rich, energy-derived, and thermally stable solid fuel, making it more suitable for advanced energy recovery applications, co-firing, and as a precursor material for activated carbon and adsorption-based environmental applications.

**Table 1:** Ultimate and proximate analysis of RDF feedstock and char.

Parameter	RDF feedstock	RDF Char (500 °C)
Proximate analysis (wt.%)		
Moisture content	10.20	1.80
Volatile matter	60.50	11.20
Fixed carbon	12.80	52.40
Ash content	16.50	34.60
Ultimate analysis (dry basis, wt.%)		
Carbon (C) %	50.40	58.20
Hydrogen (H) %	6.20	1.30
Oxygen (O) (100-(C + H + N + S)	32.10	20.80
Nitrogen (N) %	1.10	1.50
Sulphur (S) %	0.22	0.28
Chlorine (Cl) %	0.75	0.52
HHV (MJ/kg)	20.10	21.80

### Thermal analysis

**Fig. 2:**
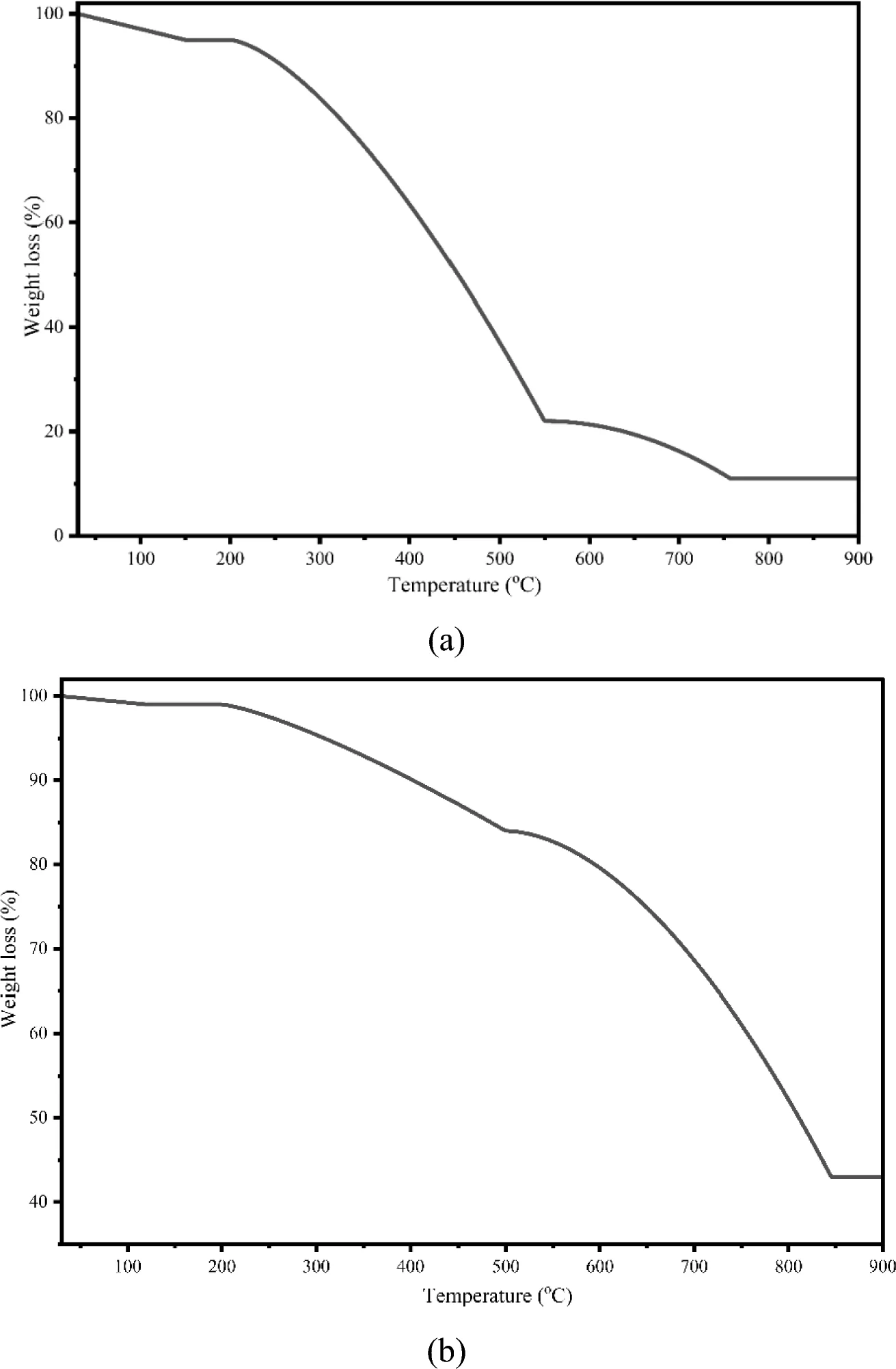
Thermogravimetric analysis of (**a**) RDF feedstock, (**b**) RDF char.

The thermal analysis of RDF feedstock and RDF char is presented in Fig. 2a,b. From Fig. 2a**,** it was noticed that the thermal decomposition behaviour and stability of the material over the temperature range of 30–900 °C. An initial minor weight loss of 5–7% is observed below 150 °C, attributed to the removal of physically adsorbed moisture and light volatile components^44^. This low-temperature loss indicates the hygroscopic nature of the precursor and confirms the presence of weakly bound water molecules. Furthermore, between 150 and 300 °C, the sample exhibits a gradual weight reduction, reaching approximately 75% of the initial mass. This stage is associated with the decomposition of unstable organic constituents such as hemicellulose and low-molecular-weight compounds^45^. The relatively slow mass loss suggests moderate thermal stability in this region. The major degradation stage occurs between 300 and 550 °C, where a sharp, pronounced weight loss is observed. Within this temperature interval, the mass decreases dramatically, resulting in a nearly 68% loss. This stage is primarily attributed to the decomposition of cellulose and lignin fractions, the breakdown of complex organic structures, and the release of volatile gases (CO, CO_2_, CH_4_, and other hydrocarbons)^44^. The maximum decomposition rate in this region indicates the material’s active pyrolytic conversion. Beyond 550 °C, the rate of weight loss slows significantly, and a gradual decline continues up to 900 °C. The residual mass stabilises at approximately 10–12%, which represents the formation of a thermally stable carbonaceous char enriched with inorganic minerals. The high residual yield at elevated temperatures indicates good char-forming ability and enhanced thermal stability, making the material suitable for high-temperature applications such as adsorbents or catalyst supports^46^.

Figure 2b confirmed the thermal degradation profile and stability of RDF char over the temperature range of 30–900 °C. An initial negligible mass loss of 1–2% below 120 °C is observed, which can be attributed to the evaporation of physically adsorbed moisture and weakly bound volatiles. The minimal loss in this region indicates low hygroscopicity and good initial thermal stability of the material^46^. Additionally, the sample exhibits a gradual weight loss, with the mass remaining above 95% from 120 to 300 °C. This stage is associated with the decomposition of light organic components and unstable functional groups, suggesting that the material retains most of its structural integrity at moderate temperatures^47^. The pronounced degradation region occurs between 300 and 550 °C, during which the weight decreases from 95 to 85%. This stage corresponds to the thermal decomposition of hemicellulose and partial degradation of cellulose, along with the release of volatile compounds. The relatively moderate mass loss in this region indicates a controlled breakdown of organic constituents rather than abrupt decomposition^48^. Major weight loss occurs at higher temperatures, particularly between 550 and 800 °C, where the mass drops sharply from 83 to 43%. This substantial loss (40%) is attributed to the decomposition of thermally stable cellulose and lignin fractions, as well as the progressive aromatisation and devolatilization of the carbon matrix^48^. The slope of the curve in this region indicates active pyrolytic reactions and material restructuring. It was noticed that above 800 °C, the weight loss becomes minimal, and a stable residue of approximately 42–44% remains at 900 °C. This high residual mass indicates significant char formation and the presence of thermally stable carbonaceous and inorganic components, confirming the suitability of the material for high-temperature applications, such as biochar-based adsorbents or catalyst supports^47^.

### FTIR analysis

**Fig. 3:**
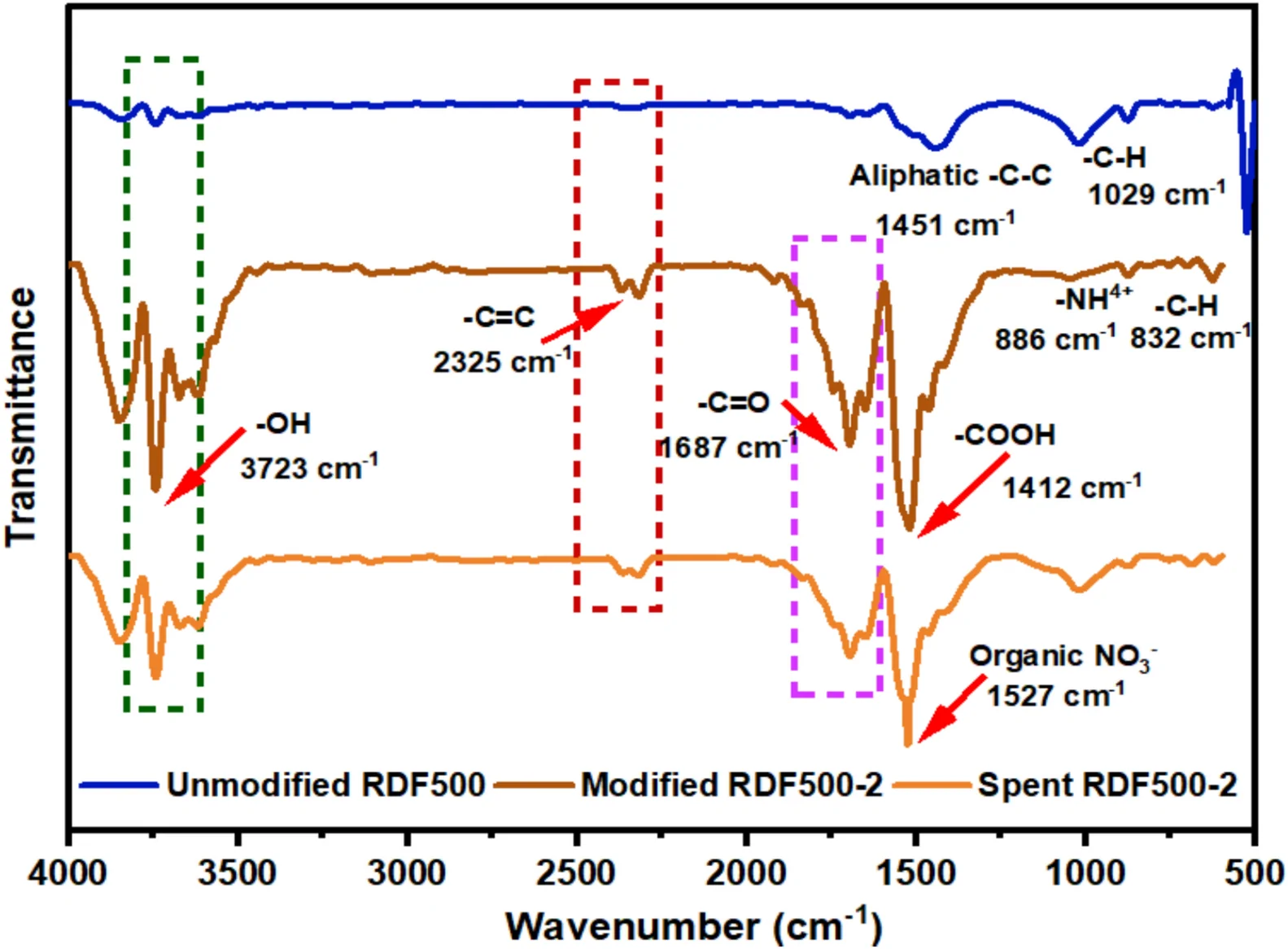
FTIR spectra of RDF char (RDF500, RDF500-2 and spent RDF500-2).

The FTIR spectra of unmodified RDF500, modified RDF500-2, and spent RDF500-2 (Fig. 3) clearly illustrate the structural and functional transformations induced by chemical modification and adsorption processes. The unmodified RDF500 sample exhibits minimal surface functionalities, with lower-intensity peaks, confirming its relatively inert carbonaceous structure, dominated by aliphatic –C–C (1451 cm^-1^) and –C–H (1029 cm^-1^) bonds^49^. Furthermore, significant spectral changes occur upon modification with zinc chloride, confirming surface activation and the formation of new oxygen-containing functional groups, which are essential for nitrate adsorption. The appearance of a strong, broad peak at 3723 cm^-1^ in the modified RDF500-2 indicates enhanced surface hydroxylation, increasing polarity and improving hydrophilicity. The peak at 2325 cm^-1^ corresponds to –C = C stretching in aromatic or unsaturated carbon chains, implying partial graphitisation of the char structure due to ZnCl_2_ activation, which promotes carbon rearrangement and enhances electron density^50^. The characteristic absorption at 1687 cm^-1^ represents the carbonyl (–C=O) stretching vibration, while the band at 1412 cm^-1^ indicates the presence of carboxylic (–COOH) groups^31^. These oxygen-rich groups serve as active binding sites for anionic nitrate species through electrostatic attraction and hydrogen bonding^50^. The peak at 1450 cm^-1^ corresponds to aliphatic –C–C stretching, while small peaks near 886 cm^-1^ and 832 cm^-1^ are attributed to -NH_4_^+^ and –C–H bending vibrations, suggesting the presence of nitrogenous functionalities incorporated during modification or pyrolysis^51^. In particular, the modified RDF500-2 spectrum shows a new band around 1500 cm^-1^, corresponding to organic nitrate (–NO₃^−^) groups, which becomes more prominent in the spent RDF500-2 spectrum after adsorption, confirming successful nitrate uptake on the adsorbent surface. This distinct peak shift and intensity increase directly indicate the interaction between surface oxygenated groups and nitrate ions during the adsorption process^52^. The presence of these nitrate-specific absorption bands in the spent RDF500-2 confirms the formation of surface complexes, such as metal-nitrate or carbon–nitrogen bonds, indicating chemisorption rather than simple physisorption. The adsorption of nitrate is mainly governed by electrostatic attraction between protonated surface functional groups and NO_3_^−^ ions, and possible weak ion-exchange interactions at active surface sites.

Furthermore, the modification process resulted in a reduction in the intensities of some bands, particularly for –C=C, –COOH and –C=O, which may be attributed to the partial consumption of reactive sites during the ZnCl_2_ impregnation and dehydration reactions that promote the condensation and cross-linking of carbon structures^33,53^. The overall reduction in transmittance intensities signifies increased surface complexity and heterogeneity. The disappearance and weakening of certain peaks after modification suggest that ZnCl_2_ acted as a dehydrating and activating agent, facilitating pore development through catalytic charring and promoting the creation of micro and mesopores. In addition, the formation of -COOZ_n_ and –C=OZ_n_ complexes after ZnCl_2_ modification explains the observed decline in –OH intensity and confirms metal–oxygen coordination, which enhances the affinity for nitrate through ion-exchange and electrostatic mechanisms^54^. The spectrum of spent RDF500-2 also indicates the persistence of –OH, –C=O, and -COOH groups, albeit with reduced intensities, suggesting that these sites participated in adsorption but were not entirely exhausted, leaving scope for regeneration^54^. The increased absorbance at 1527 cm^-1^ and broadening near 1700 cm^-1^ confirm the presence of retained nitrate species and possible hydrogen bonding with surface oxygen functionalities^54^.

The FTIR analysis clearly demonstrates that ZnCl_2_ modification substantially enhances the surface chemistry of RDF char, introducing active oxygen and nitrogen-containing functional groups that improve the affinity toward nitrate ions during adsorption. The unmodified RDF500 primarily displays a carbonaceous framework with limited reactive moieties, while the modified RDF500-2 exhibits enriched polar functionalities, improved ion-exchange potential, and chemical heterogeneity, all of which contribute to higher adsorption efficiency. The spectral changes observed after adsorption (spent RDF500-2) validate the chemical binding of nitrate onto functionalized sites via electrostatic attraction and surface complexation^50^. Consequently, FTIR results confirm the successful functionalization of RDF char through ZnCl_2_ activation and provide strong evidence of the adsorptive interaction mechanisms involved in nitrate removal, aligning with previous findings that attribute enhanced nitrate sorption to the presence of oxygenated functional groups such as –OH, –COOH, and –C = O ^1^^55^.

### BET surface area analysis

The BET surface area analysis of RDF500 and zinc chloride-modified RDF500-2 are listed in Table 2. The textural characteristics were evaluated through BET surface area, pore diameter, total pore volume, and mesopore surface area analyses. The RDF500 sample exhibited a BET surface area of 8.79 m^2^/g, whereas modification with ZnCl_2_ resulted in a slight decrease to 6.80 m^2^/g, indicating partial pore blockage due to the deposition of zinc species and the agglomeration of surface particles (Table 2**)**. It has been enhanced by the introduction of Lewis acidic Zn–O and oxygen functional groups, which form high-affinity electrostatic and inner-sphere binding sites for nitrate. Accordingly, functionalities on the surface-controlled chemisorption dominated the overall adsorption performance as compared to physical porosity. In addition, the modified RDF500-2 also demonstrated an increase in a more heterogeneous pore structure, which enhances the accessibility of adsorbate molecules during dynamic adsorption^56^. The average pore diameter of RDF500 was around 14–15 nm, which decreased after modification, confirming the formation of smaller, well-distributed pores that facilitate faster intraparticle diffusion^57^. The total pore volume of RDF500 was relatively higher than RDF500-2, suggesting that the activation process altered pore geometry by converting larger macropores into finer mesopores or micropores. This transformation is attributed to dehydroxylation and carbon matrix rearrangement induced by ZnCl_2_ activation, which promotes crosslinking and partial collapse of fragile pore walls at elevated temperatures^58^. The mesopore surface area and volume were reduced in the modified RDF500-2 sample. Due to the penetration of ZnCl_2_ (Lewis acid), the mesopore walls cause adjacent mesopores to merge into larger macropores, resulting in mesopore surface area and volume reduction. Moreover, residual ZnO species deposited within mesopore channels block pore accessibility, and ZnCl_2_ develops new microporosity by Zn volatilisation, in parallel with the consumption of carbon present in the mesopore walls^59^.

**Table 2:** Porous characterisation of RDF char.

	Unmodified char	Modified char
Property	RDF500	RDF500-2
BET surface area (m^2^/g)	8.79	6.80
Average pore diameter (nm)	15.29	5.53
Total pore volume (cc/g)	0.033	0.009
Mesopore volume (cc/g)	0.030	0.009
Mesopore surface area (m^2^/g)	7.57	3.93

**Fig. 4:**
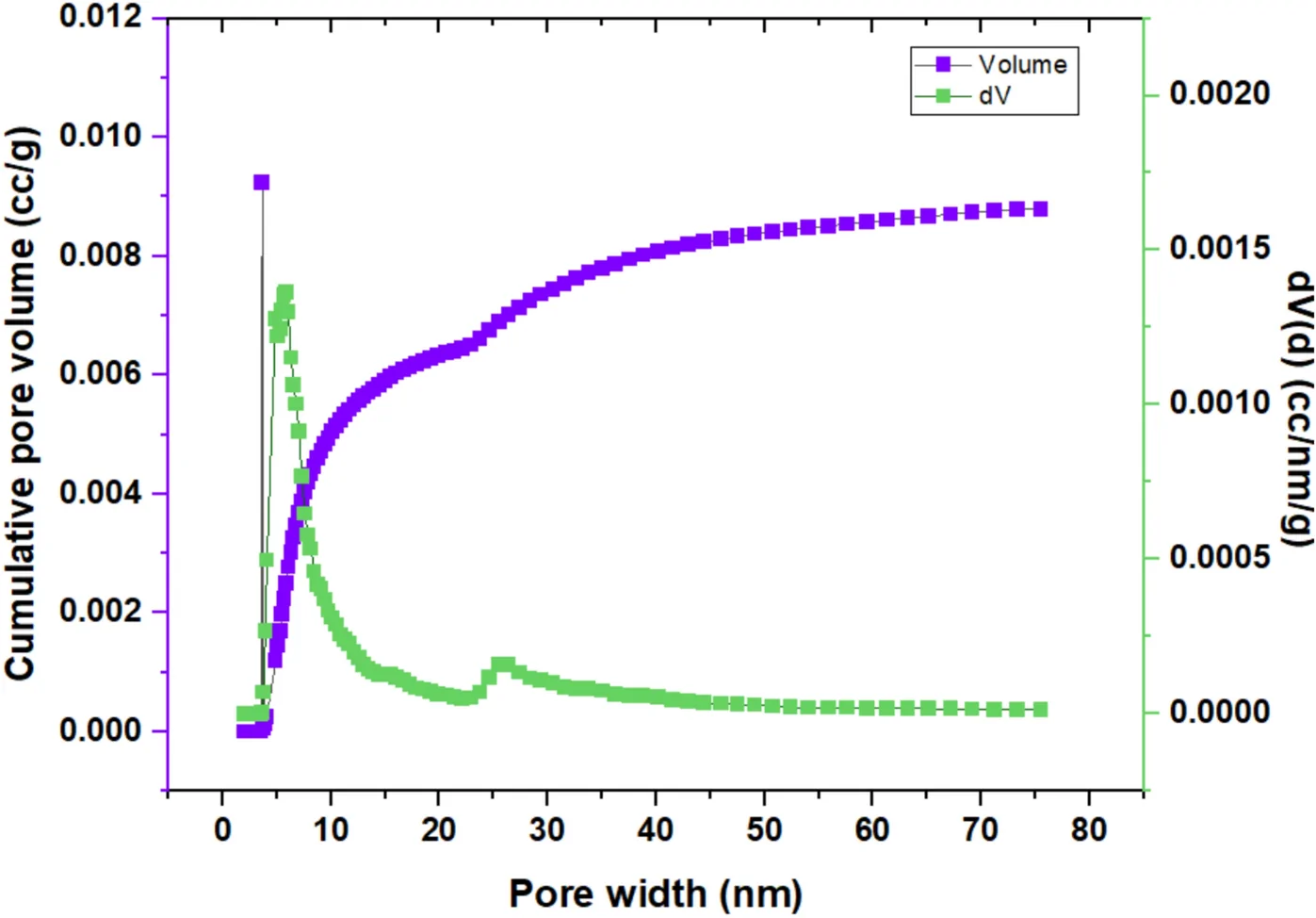
Pore size distribution of RDF500-2.

Furthermore, pore widths between 2 and 50 nm indicate that the solids are mesoporous, with a wider pore distribution in RDF500-2 (Fig. 4). Additionally, type III and type IV isotherms confirm mesoporous behaviour.

**Fig. 5:**
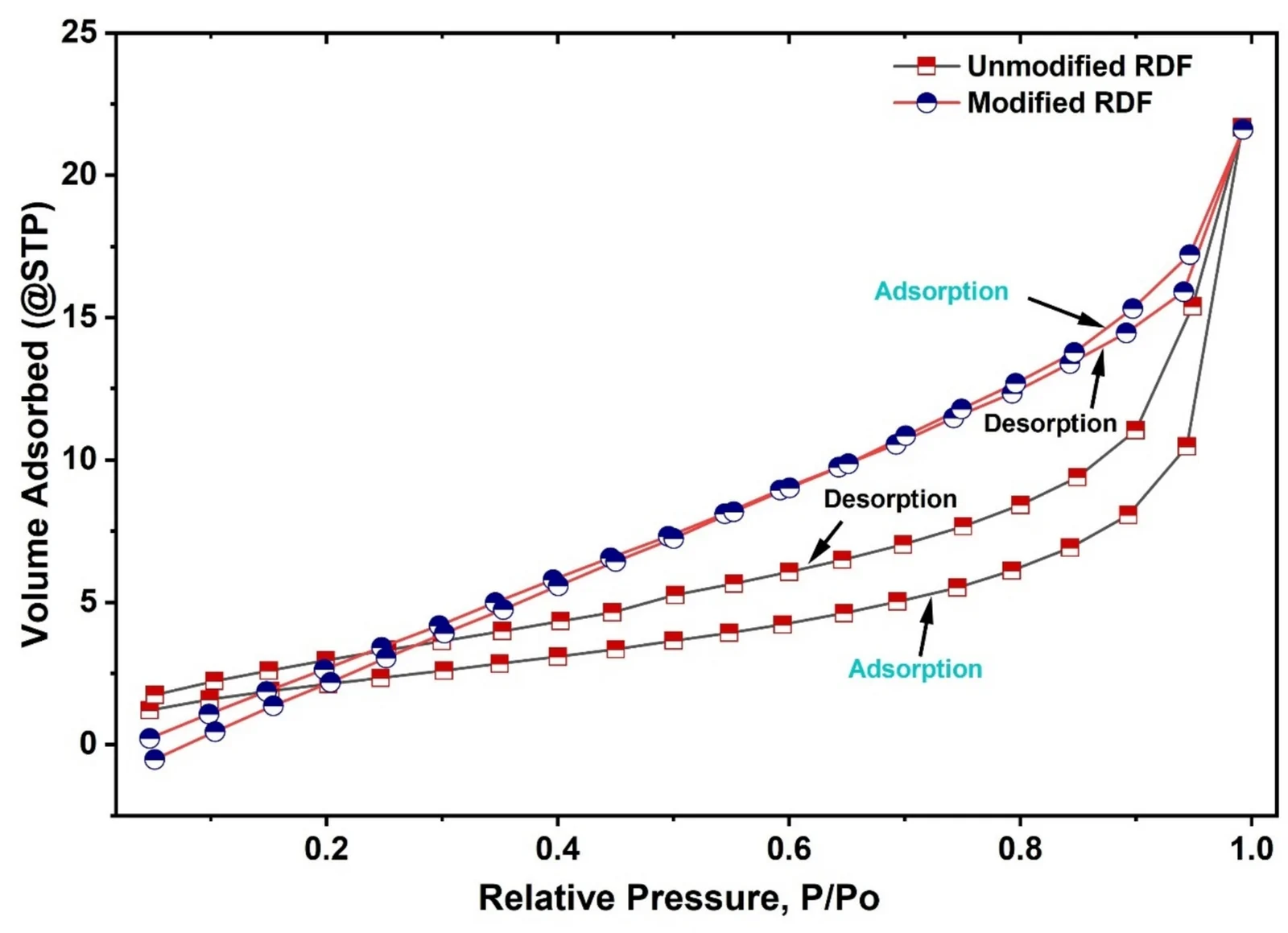
N_2_ adsorption/desorption isotherm curve of RDF500 and RDF500-2.

The mesoporous nature (2–50 nm) of RDF char is essential for adsorbing hydrated nitrate ions, as it provides a balance between surface accessibility and diffusion resistance. At relative pressures above 0.4, a clear hysteresis loop appears in the nitrogen adsorption–desorption isotherm of unmodified (RDF500) and modified (RDF500-2) char in Fig. 5**,** confirming the mesoporous nature of the sample. Unmodified RDF shows a lower adsorbed volume across the entire relative pressure range, confirming that surface modification significantly enhances adsorption capacity. Both samples exhibit Type II isotherm behaviour with H3 hysteresis loop and an early type III feature, indicating the coexistence of micro- and mesopores. The wider hysteresis loop of modified RDF indicates a greater mesopore contribution following chemical treatment. The DFT-derived pore size distribution further supports this, revealing details of the internal pore structure. At high relative pressures (P/P°), the adsorption branch shows a steep inward curve due to limited nitrogen uptake. The BET surface area of RDF char was measured as 8.79 m^2^/g. Moreover, the reduced BET area and pore volume after modification do not necessarily indicate diminished adsorption capacity; rather, they indicate surface enrichment with oxygenated groups such as –OH, –COOH, and –C=O, which provide additional electrostatic and chemisorptive interaction sites. The decline in pore volume can also be attributed to the partial filling of internal voids by ZnO complexes formed during the activation process^60^. This observation is consistent with previous findings showing that ZnCl_2_ treatment reduces the specific surface area but increases surface chemical reactivity, thereby improving adsorption efficiency toward anionic pollutants^51^. The comparison between RDF500 and RDF500-2 confirms that chemical modification has significantly transformed the surface morphology and pore distribution, producing a mesoporous, functionally rich, and chemically active adsorbent with enhanced affinity for nitrate removal, despite a marginal loss in physical surface area.

### Surface morphology analysis of char

**Fig. 6:**
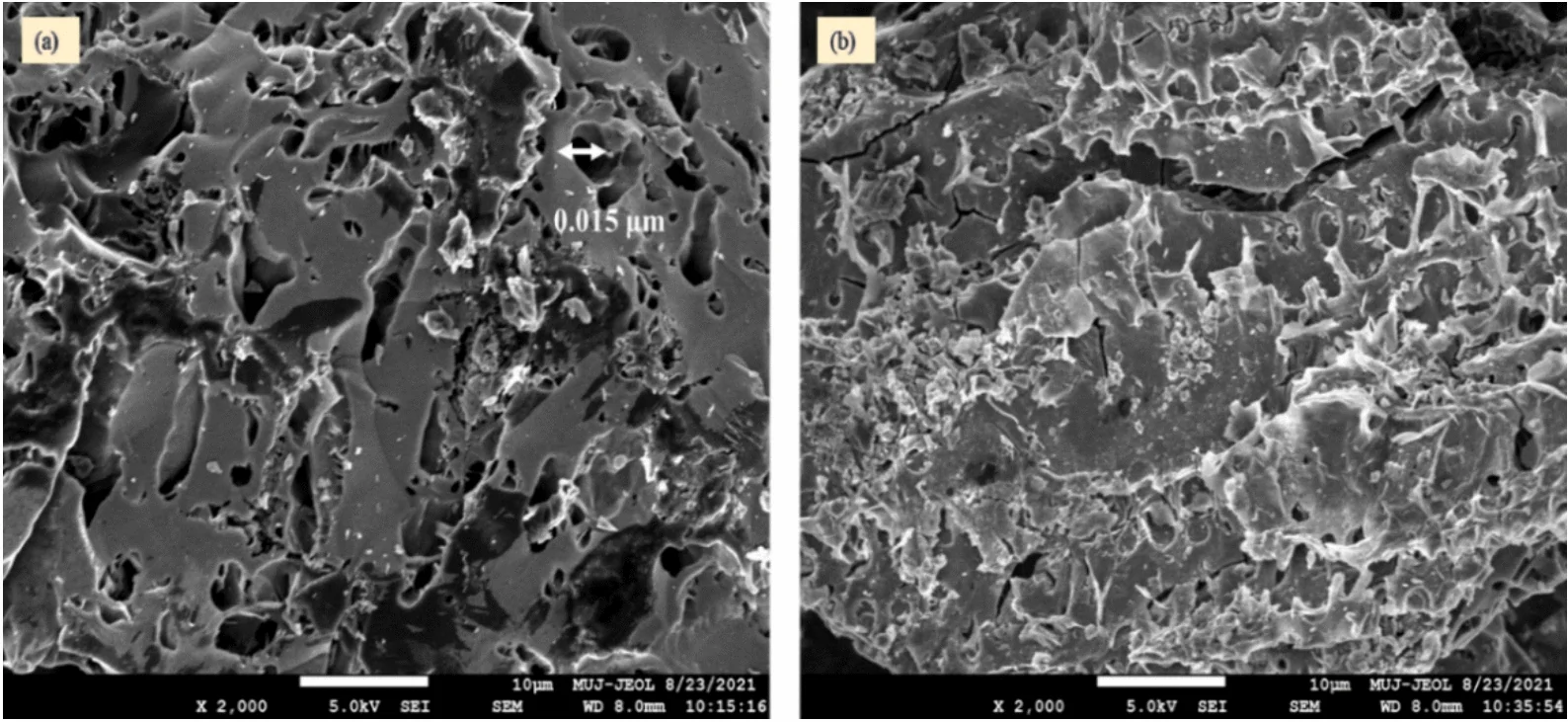
FESEM images of char samples (**a**) unmodified RDF500 (**b**) modified RDF500.

The FESEM images shown in Fig. 6a,b show the alteration of the surface structure of the char. The RDF500 char exhibits a relatively compact and less porous surface, with limited visible fissures or voids, whereas the modified RDF500 char reveals a more disturbed, roughened surface morphology with abundant micro‐cracks, lamellar fragments, and irregular voids. In RDF500, the material appears dense and consolidated, suggesting limited access to its internal pore structure. In contrast, the modified RDF500 char shows that the modification has etched or disrupted the matrix, exposing internal cavities and increasing surface roughness^61^. The measurement “0.015 µm” (15 nm) indicated by the arrow in the unmodified sample suggests detection of a fine pore or crack, though such features are sparse; in contrast, the modified sample shows many more of such features across the surface. The morphological changes in the modified RDF500 char imply that the modification process has created greater topographic heterogeneity^62^. These changes facilitate enhanced mass transfer, provide more active sites, and improve accessibility of reactants or adsorbates to internal surfaces, which is often a desirable outcome in functional carbon materials or char‐based adsorbents. The extensive disruptions (cracks, delamination) in the modified RDF500 char may also reduce diffusion resistance and shorten diffusion paths^63^. The transition from a smooth, relatively featureless surface in RDF500 to a highly rough, fractured morphology in modified RDF500 char clearly demonstrates that the modification effectively altered the char’s structure.

### Column-adsorption studies

Breakthrough curves play a crucial role in analysing the dynamic behaviour of adsorption within a column. Key parameters influencing breakthrough curve studies include the feed concentration, bed height and flow rate of adsorbate solution.

#### Effect of influent flow rate on nitrate adsorption onto RDF500-2

**Fig. 7:**
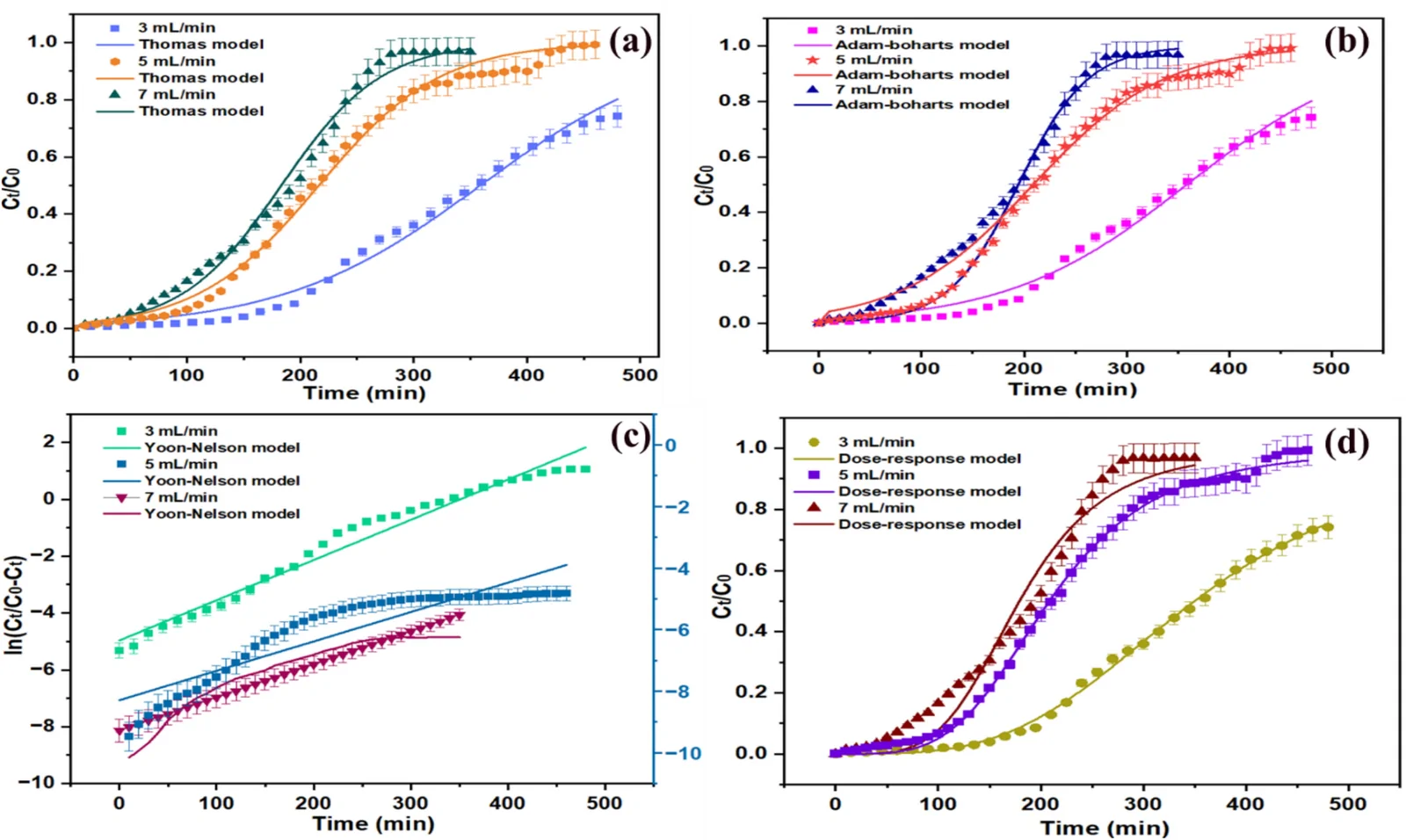
Nitrate adsorption breakthrough curve onto RDF500-2 at flow rates of 3, 5 and 7 mL/min (bed height = 10 cm, initial nitrate concentration = 128 mg/L and pH = 6.8): (**a**) Thomas, (**b**) Adam-Bohart’s, (**c**) Yoon Nelson and (**d**) Dose–response model fitting.

To examine the flow rate influences nitrate adsorption onto modified RDF char (RDF500-2), fixed-bed column studies were carried out at flow rates of 3, 5, and 7 mL/min. These tests were conducted while keeping the initial nitrate concentration and bed height constant at 128 mg/L and 10 cm, respectively. A summary of the results is provided in Table 3, and the breakthrough curves for the nitrate concentration ratio (Ct/C0) vs time (t, min) are shown in Fig. 7. The breakthrough time decreased by 41.6%, from 257 to 150 min**.** Analysis showed that initially the adsorption accelerated at lower flow rates, and in the next phase of the process, nitrate uptake decreased, resulting in reduced effectiveness. The solute residence time in the column would be longer, and breakthrough occurred later (tb increased) at lower flow rates. Furthermore, the contact time between the adsorbate and adsorbent would increase, and the adsorbent bed would have sufficient time to reach adsorption equilibrium (due to a greater availability of active sites for nitrate adsorption, which gradually decreases with increasing flow rates), thereby increasing the adsorption capacity^64^. Similarly, at higher flow rates, the residence time of solute in the column was not long enough to reach adsorption equilibrium; thus, the nitrate solution/adsorbate leaves the column before equilibrium occurs, and thus, the contact time between RDF char and nitrate is very short, resulting in a decrease in uptake capacity. At higher flow rates, the solute’s residence time in the column was insufficient to reach equilibrium for adsorption. As a result, the nitrate solution or adsorbate exits the column before equilibrium is reached, leading to very little contact time between the nitrate and RDF char and, consequently, a decrease in uptake capacity. The breakthrough curve becomes steeper, leading to decreases in both the adsorbed nitrate concentration and the breakpoint time as the flow rate increases^34,65^.

**Table 3:** Breakthrough parameters for the removal of nitrate using modified RDF char (RDF500-2).

	Q (mL/min)	C_0_ (mg/L)	Z (cm)	M (g)	t_b_ (min)	t_s_ (min)	m_in_(mg)	m _out_ (mg)	m_ad_, s (mg)	q_b_(mg/g)	q_e_(mg/g)	Y (% Removal)	SUR (g/mL)	DoSU (%)
Effect of Bed height (cm)	**5**	**128**	**5**	**6.74**	**146**	**390**	**242.23**	**132.48**	**109.75**	**11.86**	**16.02**	**45**	**10.5**	**74**
	5	128	7.5	7.88	150	340	214.19	74.18	140.01	10.85	17.77	65	10	61
	*5*	*128*	*10*	*10.65*	*175*	*460*	*291.73*	*96.9*	*194.83*	*11.92*	*18.29*	*67*	*14.4*	*65*
Effect of Initial adsorbate conc. (mg/L)	5	67	5	6.82	230	410	133.65	45.44	88.21	8.71	12.88	66	6	41
	**5**	**128**	**5**	**6.74**	**146**	**390**	**242.23**	**132.48**	**109.75**	**11.86**	**16.02**	**45**	**10.5**	**74**
	5	185	5	6.90	90	309.6	286.79	139.68	147.1	11.15	21.32	51	15.3	52
Effect of flow rate (mL/min)	3	128	10	10.65	257	480	221.44	24.31	197.13	8.53	18.51	89	13.8	41
	*5*	*128*	*10*	*10.65*	*175*	*460*	*291.73*	*96.9*	*194.83*	*11.92*	*18.29*	*67*	*14.4*	*65*
	7	128	10	10.65	150	350	313.81	155.4	158.41	11.10	14.87	50	15.8	75

The column adsorption parameters: min (mg), amount (mg), mads (mg), tb (min), t_s_ (min), qb (mg/g), q_e_ (mg/g), Y (% removal), SUR (g/mL) and DoSU (q_b_/ q_e_) were evaluated in Table 3**.** Both breakthrough (when influent concentration reaches 40 ppm) (decreases from 257 to 150 min) and exhaustion time (decreases from 480 to 350 min) were achieved earlier and nitrate uptake capacity (q_e_) and removal (Y, %) also decreased from 18.51 to 14.87 mg/g and 89 to 50% respectively, when flow rate was raised from 3 to 7 mL/min. On the other hand, the SUR (g/mL) increases from 13.8 to 15.80 g/mL with increasing flow rate (3 to 7 mL/min), due to reduced contact time and lower adsorption efficiency, as the adsorbate molecules lack sufficient time to bind effectively. Lower SUR is favourable, indicating a more efficient use of adsorbent, and higher SUR indicates that more adsorbent is consumed for the same removal. Another parameter, DoSU, quantifies the maximum utilisation of the bed, as explained by the equilibrium uptake (q_e_). In this study, the DoSU increases with increasing flow rate (0.41–0.75). There could be multiple reasons. For instance, at higher flow rates, adsorption mostly occurs at the bed surface, and the upper layers of the adsorbent rapidly utilise their capacity, while the deep layers remain underutilised, thereby increasing local sorbent utilisation.

**Fig. 8:**
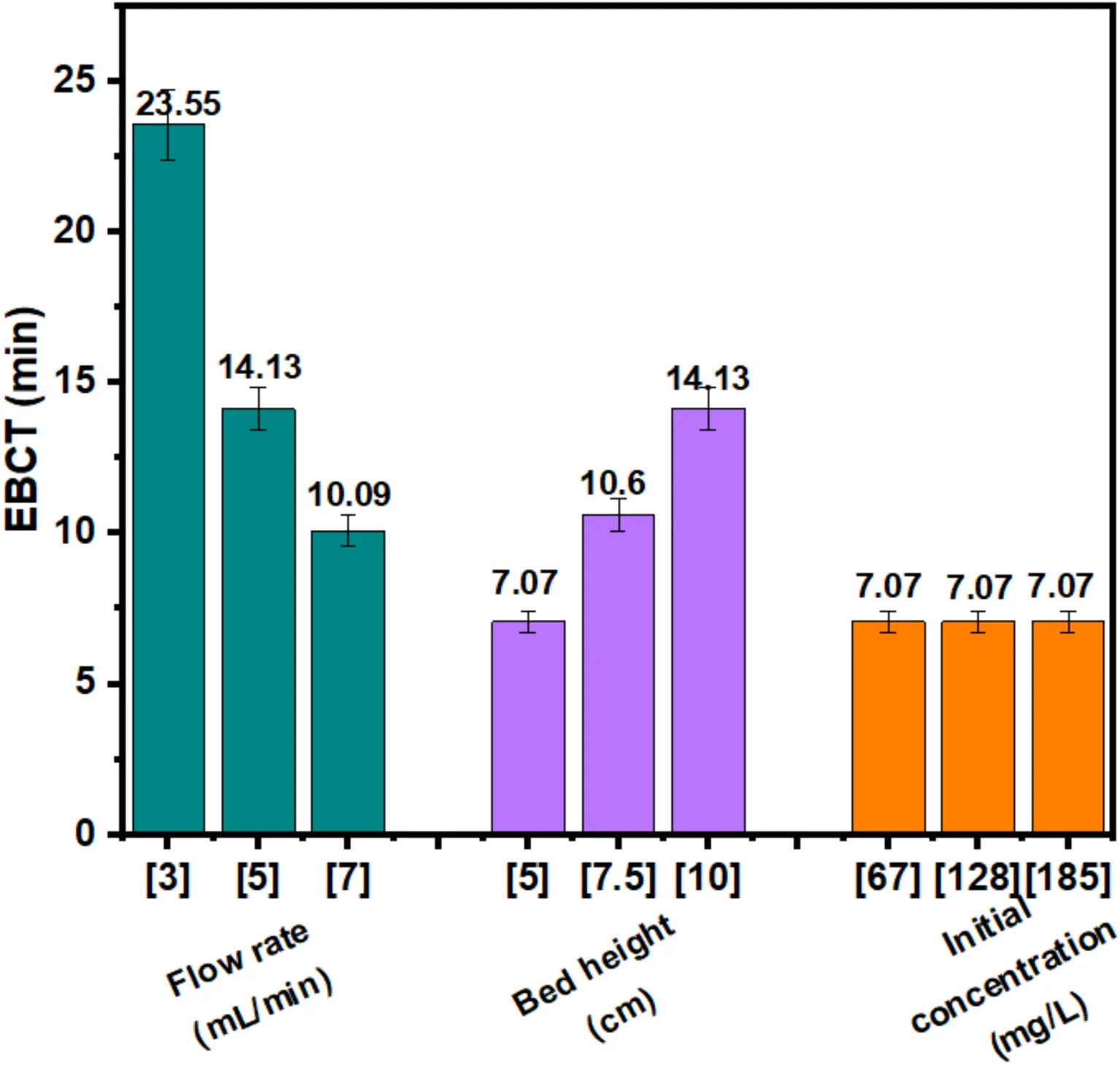
Effect of initial nitrate concentration, bed height and feed flow rate on EBCT (min).

Figure 8 illustrates the variation of empty bed contact time (EBCT) as a function of three operational parameters, flow rate, bed height, and initial concentration. These results suggest a significant influence on the efficiency of the adsorption process. From Fig. 8, it was noticed that EBCT is inversely proportional to flow rate and directly proportional to bed height. EBCT decreases markedly from 23.55 to 10.09 min as the flow rate increases from 3 to 7 mL/min. This reduction occurs because higher flow rates reduce residence time, limiting mass transfer between the adsorbate and adsorbent surfaces, and consequently lowering adsorption efficiency^66^. Conversely, the bed height increases from 5 to 10 cm, and EBCT rises from 7.07 to 14.13 min. This increase can be attributed to the longer contact pathway and the greater quantity of active sites available for adsorption, thereby enhancing solute-solid interactions^66^. Interestingly, the initial concentration (67–185 mg/L) has a negligible impact on EBCT, which remains approximately 7.07 min. This constancy indicates that EBCT is more structurally dependent on the column configuration and flow conditions than on the influent concentration. In practical terms, optimising flow rate and bed height is crucial for maximising contact time without compromising system throughput^67^.

**Fig. 9:**
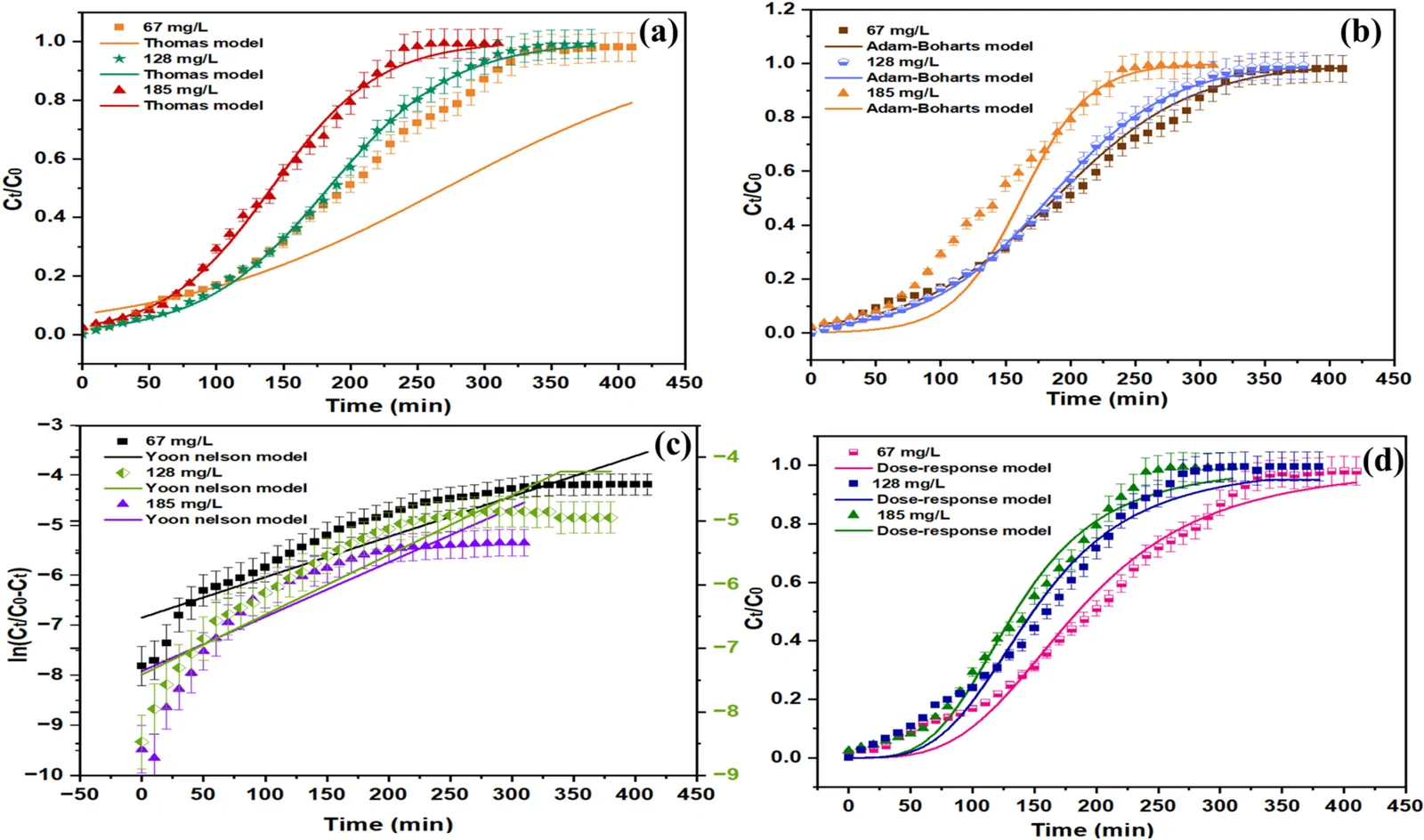
Breakthrough curve for nitrate adsorption onto RDF500-2 at different initial nitrate concentrations (67 mg/L, 128 mg/L and 185 mg/L), (Flow rate = 5 mL/min, bed height = 5 cm, pH = 6.8): (**a**) Thomas, (**b**) Adam-Bohart’s, (**c**) Yoon Nelson, and (**d**) Dose–response model fittings.

#### Effect of initial nitrate concentration

The influence of varying initial influent concentrations (67–185 mg/L) on breakthrough behaviour was systematically evaluated. The corresponding results, highlighted in Table 3 and Fig. 9, illustrate the effect of initial concentration on the shape and progression of the breakthrough curves. In this investigation, the adsorption uptake capacity increased from 12.88 to 21.32 mg/g as the initial feed (nitrate) concentration rose. The breakthrough time decreased by 60.9% from 230 to 90 min. A lower concentration leads to a late breakthrough because a smaller concentration gradient results in a slower rate of mass transfer. The concentration difference between the solute in the solution and the solute on the sorbent is the driving force. The higher adsorption capacities observed in the column fed with a high-concentration solute can be explained by the greater driving force provided by the large concentration difference. As the nitrate concentration drops from 185 to 67 mg/L, the breakthrough time rises from 90 to 230 min. With an increase in the initial nitrate concentration, SUR rises from 6 to 15.3 g/m3 due to the adsorbent’s faster saturation. DoSU initially increases from 41 to 74%, from 67% to 128 mg/L, and in the next stage of the process (from 128 to 185 mg/L), it reduces from 74 to 52%. In the present study, EBCT remains constant (7.07 min) regardless of changes in the initial nitrate concentration, as EBCT is determined by bed height (bed volume) and flow rate. However, in certain cases, a high initial concentration requires a longer EBCT, leading to early breakthrough and column exhaustion. Conversely, at lower adsorbate concentrations, EBCT may be shorter, allowing the bed sufficient time for saturation, as shown in Fig. 9. Based on literature reports for similar carbon-based and functionalized adsorbents, a reduction in adsorption capacity of approximately 20–50% can be expected in multi-ion systems, depending on the concentration and affinity of competing ions (with divalent ions like sulphate generally exerting a stronger inhibitory effect)^68,69^. Accordingly, the reported capacity of 21.32 mg/g may decrease under real water conditions, potentially in the range of 10–18 mg/g. Detailed adsorption mechanism of nitrate interaction with active sites over zinc chloride modified RDF char has already been discussed in our recent batch adsorption studies^70^.

#### Effect of bed height on nitrate adsorption onto RDF500-2

**Fig. 10:**
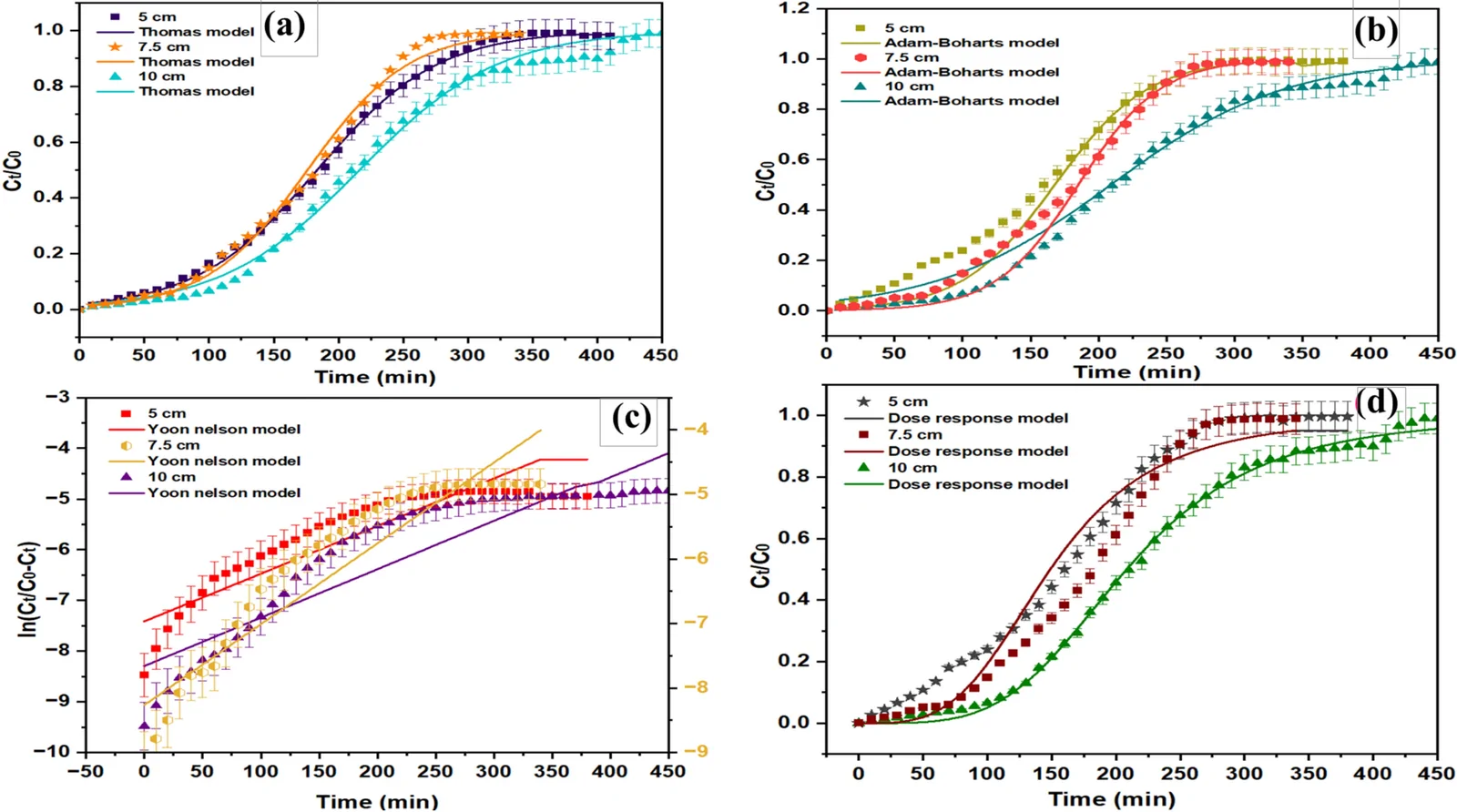
Breakthrough curve for nitrate adsorption onto RDF500-2 at different bed heights (5 cm, 7.5 cm and 10 cm), (Flow rate = 5 mL/min, initial nitrate concentration = 128 mg/L, pH = 6.8): (**a**) Thomas, (**b**) Adam-Bohart’s, (**c**) Yoon Nelson and (**d**) Dose–response model fitting.

Column-adsorption studies were conducted with different bed heights (5, 7.5, and 10 cm) at a constant flow rate of 5 mL/min and an initial nitrate concentration of 128 mg/L to investigate the effect of bed height. It was determined that both the nitrate adsorption capacity and breakthrough time increased by 19.9% (146–175 min) with increasing bed height. This is due to the reason that with an increase in bed height, a larger amount of adsorbent dose is required and provides greater adsorption reactive sites, resulting in expansion of the mass transfer zone and further increases the adsorption uptake potential (increases from 16.02 to 18.29 mg/g with an increase in bed height from 5 to 10 cm). More contact time between the RDF500-2 and the adsorbate solution results in higher nitrate removal. As bed height decreases, the breakthrough curve gets steeper. There is an increase in DoSU (61 to 65%) from 7.5 cm to 10 cm, but on the contrary, it decreases from 5 to 7.5 cm, which indicates that, due to column channelling with a bed height of 7.5 cm, the bed is not completely saturated; therefore, DoSU reduces. Axial dispersion at lower bed heights has a greater impact on column adsorption than on the actual mass transfer expected of solute with higher bed heights^64^. Additionally, the breakthrough curve exhibits an “S” shaped trend, which is the feature of a perfect system, shown in Fig. 10**.** SUR decreases with increase in bed height, as at lower bed height sorbent will fastly get wet (more contact time and better adsorbent usage) in given time and as bed height increases the adsorbent bed will be slowly used. The effects of other parameters on bed height are presented in Table 1. EBCT also increases from 7.07 to 14.13 min when the bed height is raised from 5 to 10 cm. A higher bed will influence the bed’s volume. A shorter EBCT could result in incomplete treatment because the influent moves through the media too rapidly. More EBCT signifies a greater adsorbent mass, resulting in a higher number of available sorption sites. A longer flow path increases interaction between the nitrate solution and the RDF char, thereby enhancing mass transfer and diffusion in the column.

### Breakthrough curve mathematical modelling

Comparison between the predicted and experimental breakthrough curves, as well as other parameters, was calculated using the Dose–response, Thomas, Yoon–Nelson,Adams–Bohart, and Wolborska mathematical models, with varying bed heights, flow rates, and influent nitrate concentrations (shown in Table 4). The curve between concentration ratio (Ct/C_0_) and time provides interpretations of the surface parameters of the adsorbent, its affinity and adsorption pathways^71^.

**Table 4:** The parameters of column adsorption models.

Column adsorption models	Plot	Model Parameters	Flow rate (mL/min)			Bed height (cm)			Initial nitrate concentration (mg/L)		
			3	5	7	5	7.5	10	67	128	185
Thomas model	$\frac{{C}_{t}}{{C}_{0}}$ vs t	k_Th_ (mL/mg.min)	9.18 × 10^–5^	1.5 × 10^–4^	1.7 × 10^–5^	1.70 × 10^–4^	2.02 × 10^–4^	1.5 × 10^–4^	0.4 × 10^–4^	1.7 × 10^–4^	1.35 × 10^–4^
		q_Th_ (mg/g)	12.68	12.89	15.44	17.48	14.09	12.89	12.89	17.48	18.94
		R^2^	0.988	0.985	0.99	0.986	0.998	0.985	0.988	0.986	0.966
		RMSE	0.03	0.028	0.032	0.0132	0.025	0.028	0.060	0.0132	0.071
Adam-Bohart’s model	$\frac{{C}_{t}}{{C}_{0}}$ vs t	k_BA_ (mL/mg.min)	9.14 × 10^–5^	0.000124	2.3 × 10^–4^	2.28 × 10^–4^	2.63 × 10^–4^	0.000124	2.8 × 10^–4^	2.2 × 10^–4^	2.06 × 10^–4^
		N_o_ (mg/L)	124.73	162	159.36	111.75	120.29	162	80.10	111.75	84.11
		R^2^	0.987	0.90	0.94	0.66	0.887	0.90	0.99	0.66	0.99
		RMSE	0.029	0.017	0.015	0.0079	0.010	0.017	0.0145	0.0079	0.075
Yoon–Nelson model	$\mathrm{ln}\frac{{C}_{t}}{{C}_{0}-{C}_{t}}$ vs τ	k_YN_ (min^-1^)	0.012	0.0188	0.021	0.023	0.0156	0.0188	0.0191	0.023	0.020
		τ (min)	355.05	230.20	173.41	177.81	162.56	230.20	189.40	177.81	170.05
		R^2^	0.99	0.99	1	0.99	0.99	0.99	1	0.99	0.99
		RMSE	0.769	0.397	0.684	0.224	1.132	0.397	0.276	0.224	0.428
Dose–response model	$\frac{{C}_{t}}{{C}_{0}}$ vs t	α	3.53	4.00	4.24	3.59	3.59	4.00	3.47	3.59	3.64
		β (min)	349.80	208.3	179.29	149.02	149.02	208.3	185.05	149.02	134.51
		R^2^	1	0.99	0.995	0.994	0.997	0.99	0.995	0.994	0.993
		RMSE	1.14 × 10^–2^	0.0174	5.8 × 10^–2^	6.02 × 10^–2^	0.0872	0.0174	0.294	6.02 × 10^–2^	4.98 × 10^–2^
Wolborska model	$ln\frac{{C}_{t}}{{C}_{0}}$ vs t	β_a_ (min^-1^)	0.0132	0.0169	0.0184	0.0238	0.0200	0.0128	0.0301	0.0233	0.0206
		N_0_ (mg/L)	253.50	246.05	337.22	374.33	231.96	275.14	194.13	367.36	366.73
		R^2^	1	0.95	1	1	1	1	1	1	1
		RMSE	0.908	0.749	0.688	0.550	0.703	0.894	0.682	0.551	0.77

#### Effect of flow rate

The Thomas model rate constant (K_Th_) decreased from 1.5 × 10^-4^ to 1.7 × 10^-5^ as the flow rate increased, indicating that higher flow rates shorten the residence time and reduce adsorption efficiency. Sorption capacity (q_Th_) increased from 12.68 to 15.44 mg/g, indicating that more nitrate was fed to the column inlet; however, rapid flow may have reduced the mass transfer time. The high correlation coefficients (R^2^ = 0.985 to 0.998) and low RMSE (0.028 to 0.032) show a strong fit of the Thomas model. This model performs consistently across different flow rates. The Thomas model neglects axial dispersion; however, real systems often exhibit non-ideal flow, particularly in non-uniform columns and at low flow rates, resulting in inaccurate assumptions under certain experimental conditions. Intraparticle diffusion, which is significant in porous materials and is associated with non-linear adsorption isotherm behaviour close to equilibrium, was neglected. Also limits itself in multicomponent systems. The Thomas model was observed to be appropriate for systems in which the limiting step is not internal or external adsorption diffusion. It was observed that theAdams–Bohart coefficient increases from 9.14 × 10^-5^ to 2.3 × 10^-4^, indicating that higher flow rates promote adsorption at the initial stage but subsequently limit it at later stages. No increase was observed, and an elevated value was seen at 5 mL/min, indicating that a higher flow rate utilises more sites initially. Despite having high R^2^ (0.90 to 0.987), only the initial portion of the breakthrough curve (Ct < 45 ppm, nitrate permissible limit) was well-fitted by theAdams–Bohart model^72^. TheAdams–Bohart model exhibits a poor fit at adsorption equilibrium and saturation, as well as lower bed heights and flow rates. This model excels at rapid parameter estimation.

The Yoon–Nelson model assumes that the probability of adsorption is directly related to breakthrough. The YN rate constant, k_YN_, increased from 0.012 to 0.021 as the feed nitrate flow rate increased. The time required to reach the breakthrough point (C_t_ = 40 ppm), (τ) decreased sharply from 355.05 min to 173.41 min as influent nitrate flow rates increased from 3 to 5 mL/min, indicating a faster breakthrough. The error (ε) between experimental and predicted values was in the range 39.7 to 76.9% with higher correlation coefficients, R^2^ of (0.99–1), with the rate of operation of flow rates employing the Yoon–Nelson model^72^. The reason was the adsorbent’s earlier saturation, which led to faster nitrate transfer. It predicts 50% of the breakthrough time. This model has limitations, as it does not directly calculate adsorption capacity. Dose–Response adsorption rate, α increased from 3.53 to 4.24, and β decreased (349.80 to 179.29 min), indicating a decrease in breakthrough time and higher adsorption capacity. Excellent model fit (R^2^ > 0.99), with a slight increase in RMSE. The D-R model is typically used for S-shaped breakthrough curves. According to the Wolborska model, the external mass transfer kinetic coefficient (βa) increased from 0.0132 to 0.0184 min^-1^ as the flow rate rose from 3 to 7 mL/min. which results in a decrease in external film resistance. Higher flow rates reduce the boundary layer thickness, thereby enhancing external mass transfer, which suggests that diffusion is not the limiting factor^73^. The correlation coefficient (R^2^ = 0.95–1) and RMSE (0.68–0.90) suggest that this model is not a good fit to the experimental data across varying flow rates.

#### Effect of bed height

With an increase in the bed height of an adsorbent, k_Th_ (Thomas rate constant) increases from 1.7 × 10^-5^ to 1.8 × 10^-4^, resulting in more adsorption zones with better contact time and higher adsorption uptake capacity. In contrast, the qe decreases after 7.5 cm, suggesting saturation or mass-transfer limitations at larger bed heights. Although there was no direct relation between k_Th_ and q_e_ at different bed heights. Despite having a good R^2^ value (0.9850–0.9900) and a lower RMSE value of 1.32–2.80%, Thomas’ model is not a good fit for varying bed heights. In theAdams–Bohart model, the kBA increases, indicating higher kinetics. N_0_ increase in bed height, indicating better adsorption zone distribution. As bed height and volume increase, the time needed for Ct/C_0_ = 50% (τ) increased from 177.81 to 230.20 min. The Yoon–Nelson model exhibited strong correlations (R^2^ = 0.99 and 1) at various bed heights, with increasing YN model constant. In the D-R model, with an increased bed height from 5 to 10 cm, β increases (resulting in a longer breakthrough time) from 149.02 to 208.3 min, and α increases, suggesting enhanced adsorption efficiency. R^2^ remains high^34^. The results indicate that a smaller bed height leads to early breakthrough and lower capacity, and a constant β indicates an insufficient increase in bed mass. This model yields the best fit with a correlation coefficient, R^2^ of 0.99. Wolborska’s model distinguishes between film and internal diffusion at varying bed heights, confirming that external resistance is the least effective parameter in this case. From Table 4**,** it was observed that the mass transfer coefficient βa and N_0_ increase with increasing overall adsorption capacity at higher bed heights.

#### Effect of initial adsorbate concentration

The Thomas model rate constant (K_Th_) decreased from 1.4 × 10^-4^ to 1.35 × 10^-5^ as influent concentration increased, which implies that lower initial nitrate concentration reduces the residence time, further reducing adsorption efficiency, and the sorption uptake (qe) rose from 12.89 to 18.94 mg/g. A steeper curve and quicker breakthrough time are the results of treating that smaller solution with a higher starting nitrate content. It was determined that lower q_e_ values will result from higher effluent concentrations. As the influent concentration rises, binding sites are quickly saturated, and the driving force increases, decreasing the mass transfer zone where adsorption occurs^74^. The same trend (as initial nitrate concentration increased, theAdams–Bohart rate constant (K_BA_) decreased, whereas the adsorption capacity (q_e_) increased according to the Thomas model) was examined for theAdams–Bohart model^72^. The resistance to mass transfer increases with higher nitrate concentrations, leading to a decrease in the rate constants from 2.8 × 10^-4^ to 2.06 × 104. Additionally, as the influent nitrate concentration increased, so did the saturation concentration N_o_.

In contrast, the Yoon–Nelson rate constant (K_YN_) was found to increase with higher influent nitrate concentrations, consistent with findings by^71^. According to the Yoon–Nelson model, the time required for the effluent concentration to reach 50% of the influent (τ) decreased from 189.40 to 170.05 min with an increase in nitrate concentration from 67 to 185 mg NO_3_^-^/L. However, the percentage error (ε) between the experimentally determined and model-predicted τ values ranged from 27.6 to 42.80%. These discrepancies indicate that the Yoon–Nelson model does not accurately represent the experimental data across varying initial nitrate concentrations, limiting its reliability and applicability.

With an increase in initial nitrate concentration from 67 to 185 mg/L, β decreases (earlier breakthrough) from 185.05 to 134.51 min, while α increases from 3.47 to 3.59, resulting in a steeper breakthrough in the Dose–Response model. Despite a higher concentration, the model fits the experimental data well (R^2^ = 0.99). The results show that, even though the breakthrough is delayed, the curve broadens at low α, indicating that, rather than a sharp transition from adsorption to exhaustion, the bed undergoes a gradual saturation process. This indicates the presence of more non-uniform adsorption sites and slower nitrate diffusion within the pores. At higher nitrate concentrations, breakthrough occurs earlier, resulting in a sharper curve and an improved RMSE (2.94 to 6.02%).

From Table 4, it was observed that as the initial nitrate concentration increased from 67 to 185 mg/L, the driving force for diffusion also increased, thereby enhancing the adsorption rate. The value of β_a_ decreases from 0.0301 to 0.0206 min^-1^. In the present study, the Wolborska model was used to determine whether film diffusion resistance was overcome by an increased driving force, with respect to bed height. It indicated whether mass transfer has been sufficiently improved to meet the growing adsorption demand. The value of N_0_ increases from 194.13 to 366.73 mg/L because more active sites on the adsorbent particles are available for nitrate sorption at higher initial nitrate concentrations^73^.

Thomas’ model provided a good fit across a range of initial concentrations and flow rates. According to the Langmuir isotherm assumption, nitrate adsorption occurs in a monolayer with no axial dispersion, as indicated by the high agreement between the experimental data and the Thomas model. The D-R model fits best in all cases, indicating that it is most suitable for curve fitting and empirical design when other models show a poor fit. The Adam-Bohart model did not fit well in any of the cases, which indicates that internal diffusion takes place during the early breakthrough of adsorption^75^. The YN model provided the best fit for the initial adsorbate concentration. Yoon–Nelson and Thomas’ model provides a good prediction of the dynamic behaviour of RDF char^67^.

The applicability of the Wolborska model enables design optimisation, kinetic analysis, and scale-up. Also, the model estimates that the external mass transfer coefficient βa accounts for the performance at various initial adsorbate concentrations, flow rates, and bed depths applicable to systems with short contact times. The predicted and experimental breakthrough curves were highlighted in Fig. S3. It was found that the Wolborska model possesses good correlation with experimental values. Wolborska model was fitted only to the initial portion of the breakthrough curve (low Ct/C_0_ region, typically Ct/C_0_ < 0.05–0.2), In this narrow range, the data points exhibit near-linear behaviour, which can result in very high R2 values despite a limited number of data points. However, when RMSE is calculated over the same dataset, even small deviations become significant, leading to comparatively higher RMSE values. Therefore, the high R2 does not necessarily indicate a good overall fit across the entire breakthrough curve but rather reflects localised linear fitting in the initial region. Further, the model is described as less suitable for full breakthrough prediction compared to other models. The Wolborska model explains the external mass transfer mechanisms governing the initial stage of adsorption; however, for a comprehensive evaluation of column performance, it should be applied in conjunction with models such as the Thomas and Dose–Response models, which more effectively analyse the complete breakthrough behaviour under different flow conditions. This indicates that these models can be used together for pilot-scale column design. They help accurately estimate the saturation time and the mass transfer zone. Table 5. summarises the findings from different studies on how feed flow rate, initial nitrate concentration and bed height affect adsorption column performance. All four studies by^65,71,76,77^ report the consistency in the trend. The RDF char utilised in this study exhibits properties similar to those of neem leaf powder (adsorbent) reported by^67^ and is correlated with jackfruit leaf powder, utilised for dye removal^65^. The trend observed in most studies was uniform: adsorption capacity increased with reduced flow rate, increased bed height, and increased initial nitrate concentration. These parameters are critical for scale-up and design optimisation in fixed-bed column processes, providing a better understanding of efficiency and operational costs. Additionally, it provides insights into how each factor affects adsorption by predicting various mathematical models.

**Table 5:** Comparative analysis of adsorption performance under different influent conditions and column configurations. [Upward arrow- Increasing, downward arrow—decreasing].

Effect of influent flow rate, initial concentration and bed height			References
Flow rate ↑ Ads. Capacity ↓ Breakthrough time ↓	Initial adsorbate conc ↑ Ads. Capacity ↑ Breakthrough time ↓	Bed ht ↑ Ads. Capacity ↑ Breakthrough time ↑	^65^
Flow rate ↑ Ads. Capacity ↓ Breakthrough time ↓	Initial adsorbate conc ↑ Ads. Capacity ↑ Breakthrough time ↓	Bed ht ↑ Ads. Capacity ↑ Breakthrough time ↑	^75^
Flow rate ↑ Ads. Capacity ↓ Breakthrough time ↓	Initial adsorbate conc ↑ Ads. Capacity ↑ Breakthrough time ↓	Bed ht ↑ Ads. Capacity ↑ Breakthrough time ↑	^34^
Flow rate ↑ Ads. Capacity ↓ Breakthrough time ↓	Initial adsorbate conc ↑ Ads. Capacity ↑ Breakthrough time ↓	Bed ht ↑ Ads. Capacity ↑ Breakthrough time ↑	^78^
Flow rate ↑ Ads. Capacity ↓ Breakthrough time ↓	Initial adsorbate conc ↑ Ads. Capacity ↓ Breakthrough time ↓	Bed ht ↑ Ads. Capacity ↓ Breakthrough time ↑	^79^
Flow rate ↑ Ads. Capacity ↓ Breakthrough time ↓	Initial adsorbate conc ↑ Ads. Capacity ↑ Breakthrough time ↓	Bed ht ↑ Ads. Capacity ↑ Breakthrough time ↑	^80^
Flow rate ↑ Ads. Capacity ↓ Breakthrough time ↓	Initial adsorbate conc ↑ Ads. Capacity ↓ Breakthrough time ↓	Bed ht ↑ Ads. Capacity ↑ Breakthrough time ↑	^74^
**Flow rate- constant**	Initial adsorbate conc ↑ Ads. Capacity ↑ Breakthrough time ↓	Bed ht ↑ Ads. Capacity ↑ Breakthrough time ↑	^81^
Flow rate ↑ Ads. Capacity ↓ Breakthrough time ↓	Initial adsorbate conc ↑ Ads. Capacity ↓ Breakthrough time ↓	Bed ht ↑ Ads. Capacity ↑ Breakthrough time ↑	^76^
Flow rate ↑ Ads. Capacity ↑ Breakthrough time ↑	Initial adsorbate conc ↑ Ads. Capacity ↑ Breakthrough time ↓	Bed ht ↑ Ads. Capacity ↑ Breakthrough time ↑	^82^
Flow rate ↑ Ads. Capacity ↓ Breakthrough time	Initial adsorbate conc ↑ Ads. Capacity ↑ Breakthrough time ↓	Bed ht Ads. Capacity Breakthrough time	^71^
Flow rate ↑ Ads. Capacity ↓ Breakthrough time ↓	**Initial adsorbate conc.- constant**	Bed ht ↑ Ads. Capacity ↑ Breakthrough time ↑	^83^
Flow rate ↑ Ads. Capacity ↓ Breakthrough time ↓	Initial adsorbate conc ↑ Ads. Capacity ↑ Breakthrough time ↓	Bed ht ↑ Ads. Capacity ↑ Breakthrough time ↑	^64^
Flow rate ↑ Ads. Capacity ↓ Breakthrough time	Initial adsorbate conc ↑ Ads. Capacity ↑ Breakthrough time ↓	Bed ht ↑ Ads. Capacity ↑ Breakthrough time ↑	^77^
Flow rate ↑ Ads. Capacity ↓ Breakthrough time ↓	Initial adsorbate conc ↑ Ads. Capacity ↑ Breakthrough time ↓	Bed ht ↑ Ads. Capacity ↑ Breakthrough time ↑	^72^
Flow rate ↑ Ads. Capacity ↓ Breakthrough time ↓	**Initial adsorbate conc.-constant** ↓	Bed ht ↑ Ads. Capacity ↑ Breakthrough time ↑	^84^
Flow rate ↑ Ads. Capacity Breakthrough time	Initial adsorbate conc ↑ Ads. Capacity ↑ Breakthrough time ↓	Bed ht ↑ Ads. Capacity ↑ Breakthrough time ↑	^36^
Flow rate ↑ Ads. Capacity ↓ Breakthrough time ↓	Initial adsorbate conc ↑ Ads. Capacity ↑ Breakthrough time ↓	Bed ht ↑ Ads. Capacity ↑ Breakthrough time ↑	^85^
Flow rate ↑ Ads. Capacity ↓ Breakthrough time ↓	Initial adsorbate conc ↑ Ads. Capacity ↑ Breakthrough time ↓	Bed ht ↑ Ads. Capacity ↑ Breakthrough time ↑	Present study

### Analysis of column and batch adsorption

**Fig. 11:**
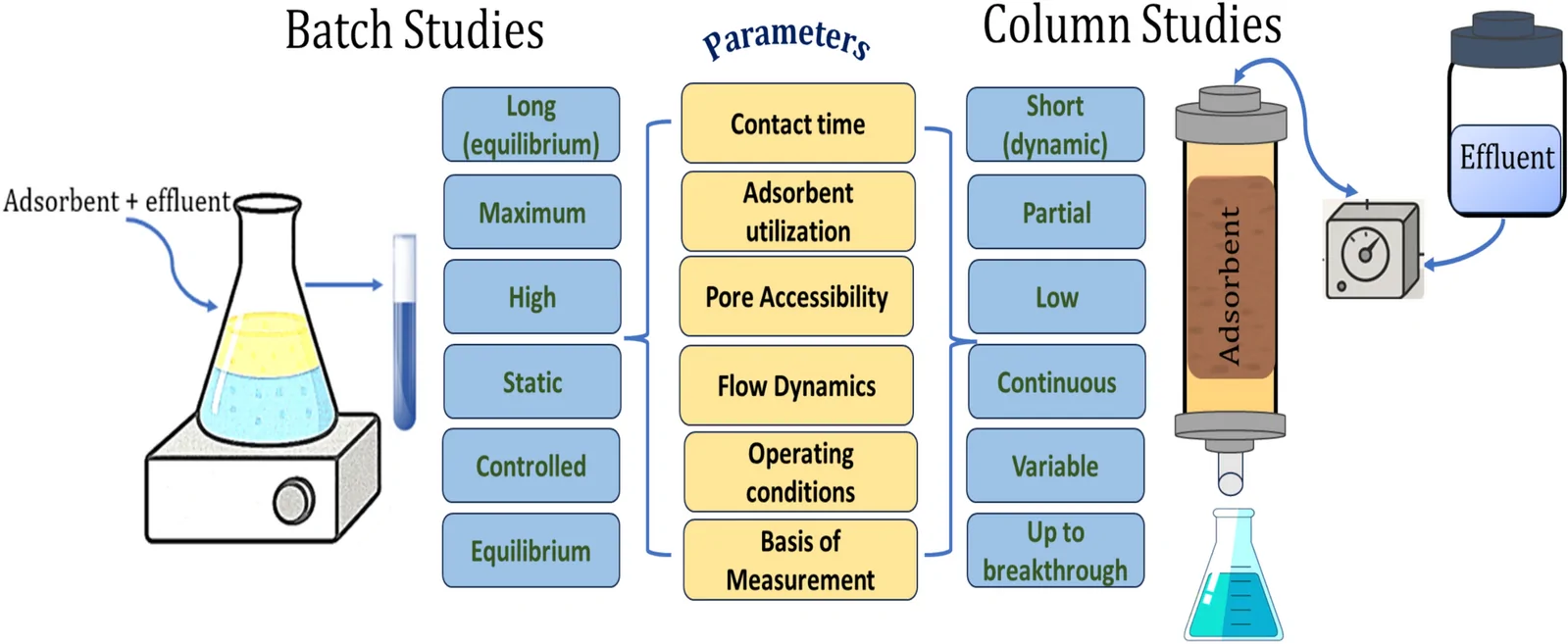
Batch and column adsorption studies.

The significant difference between the adsorption capacities of RDF500-2 obtained in batch adsorption studies (160 mg/g)^70^ and fixed-bed column studies (21.32 mg/g) can be attributed to several key technical factors. Detailed analyses are highlighted in Fig. 11. In batch systems, the conditions are optimised to reach equilibrium, allowing sufficient contact time for the adsorbate to ensure thorough interaction with the adsorbent surface^33^. This maximises the utilisation of both external and internal (microporous and mesoporous) surfaces of RDF500-2. In contrast, fixed-bed columns operate under dynamic, non-equilibrium conditions, where the adsorbate flows continuously through the bed, resulting in insufficient contact time and inadequate utilisation of adsorption sites. Mass transfer limitations also play a major role in external film diffusion^81^ more relevant in column setups due to the absence of agitation and the presence of flow resistance, resulting in reduced adsorption efficiency. Additionally, factors such as higher flow rates, bed height-to-diameter ratios, and empty bed contact time (EBCT = 14.13 min), and even channelling, lead to the formation of a finite mass transfer zone (MTZ) where only a portion of the bed is effectively utilised before breakthrough and therefore limits adsorption^73^. Additionally, flow conditions favour external mass transfer but limit pore diffusion, confining adsorption to more accessible sites. Thus, the observed difference is governed by diffusion resistance, MTZ development, and non-equilibrium operation. The batch system benefits from uniform conditions such as temperature and pH, whereas gradients in these parameters may develop along the fixed-bed column^76^. Moreover, batch experiments are often conducted with higher and stable initial adsorbate concentrations and optimised adsorbent dosages, yielding higher capacity. In column studies, adsorption capacity is typically calculated only up to breakthrough time, which does not accurately reflect the adsorbent’s equilibrium saturation potential. These combined operational differences mean that the 160 mg/g capacity observed in batch studies represents a theoretical maximum under ideal laboratory-scale conditions, while the 21.32 mg/g from fixed-bed columns reflects the more constrained, practically relevant performance of the system. This gap highlights the need to scale up and optimise column parameters to bridge the gap between lab-scale potential and real-world performance of modified RDF char (RDF500-2) as an adsorbent.

#### Column scale-up designing parameters and cost analysis

For adsorption column scale-up design, column length, adsorbent mass, bed volume, and residence time, including bulk density and surface area, are crucial parameters (Table 6 provides the lab-scale studies and parameters after column scale-up) to be considered. Stepwise scale-up and cost analysis of biochar derived from RDF has been proposed. The fixed-bed column system was designed and scaled up from laboratory to industrial scale utilizing RDF char to achieve 80% of removal efficiency with basis of 100 L/min of flow rate (scaling-up to approx. 20,000 times), at 1300 mg/L of initial nitrate concentration (C_0_) with scaling-up of parameters such as mass of adsorbent, column length (cm), volume of the bed (cm^3^), adsorption capacity (mg/g), mass of nitrate adsorbed (mg) and residence time (min), while maintaining an EBCT of 14.13 min. The influent concentration of 1300 mg/L (actual groundwater conc.) was selected based on the objective to treat highly contaminated groundwater reported in certain regions (Churu, Jaisalmer and parts of Rajasthan, India), thereby ensuring a conservative design basis. The column experiments within the concentration range 67–185 mg/L calibrated the process model and established kinetic and equilibrium parameters that could then be applied to higher-concentration predictions (scale-up). The adsorption process may exhibit non-linear behaviour during large-scale transitions (20,000-scale-up fold) due to factors such as axial dispersion, mass transfer limitations, and hydrodynamic variations. The scale-up design steps were proposed based on a constant empty bed contact time (EBCT), a similar bed porosity (ε = 0.89), and a consistent particle size (1.5 mm). Based on these parameters, the hydrodynamic and mass-transfer conditions were assumed to remain comparable between the lab-scale and the scaled-up systems. Moreover, the design ensures an appropriate L/D ratio (6), widely accepted for minimising channelling and ensuring uniform flow distribution. Further validation through pilot-scale studies and advanced modelling would be necessary to fully capture non-linear scale-up effects in future work. Equations, the designing steps, and detailed calculations for scale-up have been provided in the supplementary file.

**Table 6:** Column parameters.

Column parameters	Mathematical correlation	Value	Value (after scale-up)
Required bed height (Z), cm	$\mathrm{B}\mathrm{e}\mathrm{d} \mathrm{V}\mathrm{o}\mathrm{l}\mathrm{u}\mathrm{m}\mathrm{e}, {\mathrm{V}}_{\mathrm{b}}=\uppi {\mathrm{r}}^{2}Z$ $\frac{\mathrm{L}}{\mathrm{D}}=6$, (L = Z)	10	400
Column diameter, D (cm)	2r (r is radius)	3	67.08
Flow rate (Q), L/min	Scaled up (assumed)	5	100 (basis)
Required bed volume, V_b_ (cm^3^)	$\mathrm{B}\mathrm{e}\mathrm{d} \mathrm{v}\mathrm{o}\mathrm{l}\mathrm{u}\mathrm{m}\mathrm{e} ({\mathrm{V}}_{\mathrm{b}})=\mathrm{Q} \mathrm{x} \mathrm{E}\mathrm{B}\mathrm{C}\mathrm{T}$ $\mathrm{B}\mathrm{e}\mathrm{d} \mathrm{V}\mathrm{o}\mathrm{l}\mathrm{u}\mathrm{m}\mathrm{e}, {\mathrm{V}}_{\mathrm{b}}=\uppi {\mathrm{r}}^{2}\mathrm{Z}$		1413 × 10^3^
Required adsorption Capacity (q_e_), mg/g	${q}_{e}=\frac{{\mathrm{m}}_{\mathrm{a}\mathrm{d},{\mathrm{t}}_{\mathrm{s}}}}{M}$	18.29	18.29
Bulk density, ρ_b_ (g/cm^3^) Particle density, ρ_p_ (g/cm^3^)	${\mathrm{B}\mathrm{u}\mathrm{l}\mathrm{k} \mathrm{d}\mathrm{e}\mathrm{n}\mathrm{s}\mathrm{i}\mathrm{t}\mathrm{y}, \uprho }_{\mathrm{b}}=\frac{\mathrm{M}\mathrm{a}\mathrm{s}\mathrm{s}}{\mathrm{V}\mathrm{o}\mathrm{l}\mathrm{u}\mathrm{m}\mathrm{e}}$ $\mathrm{P}\mathrm{a}\mathrm{r}\mathrm{t}\mathrm{i}\mathrm{c}\mathrm{l}\mathrm{e} \mathrm{d}\mathrm{e}\mathrm{n}\mathrm{s}\mathrm{i}\mathrm{t}\mathrm{y}, {\uprho}_{\mathrm{p}}=\frac{\mathrm{T}\mathrm{o}\mathrm{t}\mathrm{a}\mathrm{l} \mathrm{M}\mathrm{a}\mathrm{s}\mathrm{s} \mathrm{o}\mathrm{f} \mathrm{s}\mathrm{a}\mathrm{m}\mathrm{p}\mathrm{l}\mathrm{e}}{\mathrm{V}\mathrm{o}\mathrm{l}\mathrm{u}\mathrm{m}\mathrm{e} \mathrm{d}\mathrm{i}\mathrm{s}\mathrm{p}\mathrm{l}\mathrm{a}\mathrm{c}\mathrm{e}\mathrm{d}}$ $\mathrm{P}\mathrm{o}\mathrm{r}\mathrm{s}\mathrm{o}\mathrm{i}\mathrm{t}\mathrm{y}, \upvarepsilon =1 - \frac{{\uprho}_{\mathrm{b}}}{{\uprho}_{\mathrm{p}}}=1- \frac{\mathrm{B}\mathrm{u}\mathrm{l}\mathrm{k} \mathrm{d}\mathrm{e}\mathrm{n}\mathrm{s}\mathrm{i}\mathrm{t}\mathrm{y}}{\mathrm{P}\mathrm{a}\mathrm{r}\mathrm{t}\mathrm{i}\mathrm{c}\mathrm{l}\mathrm{e} \mathrm{d}\mathrm{e}\mathrm{n}\mathrm{s}\mathrm{i}\mathrm{t}\mathrm{y}}$	0.15	0.15 1.32 0.89
Amount of nitrate adsorbed (g)	${\mathrm{m}}_{\mathrm{a}\mathrm{d},{\mathrm{t}}_{\mathrm{s}}}= {q}_{e.}.M$	-	3876.56
Removal efficiency (Y), %	$Y=\frac{{\mathrm{m}}_{\mathrm{a}\mathrm{d},{\mathrm{t}}_{\mathrm{s}}}}{{\mathrm{m}}_{\mathrm{i}\mathrm{n},{\mathrm{t}}_{\mathrm{s}}}}=\frac{{\mathrm{m}}_{\mathrm{a}\mathrm{d},{\mathrm{t}}_{\mathrm{s}}}}{{C}_{0}Q{t}_{s}}$	67 65 (DoSU, %)	80% -
Saturation time, ts (min) Residence time, RT (min)	${\mathrm{t}}_{\mathrm{s}}=\frac{{\mathrm{m}}_{\mathrm{i}\mathrm{n},{\mathrm{t}}_{\mathrm{s}}}}{{C}_{0}Q}$ $RT=\frac{{\mathrm{V}}_{\mathrm{b}} x \varepsilon }{\mathrm{Q}}= \frac{Bed volume x porosity of \mathrm{b}\mathrm{e}\mathrm{d}}{\mathrm{F}\mathrm{l}\mathrm{o}\mathrm{w} \mathrm{r}\mathrm{a}\mathrm{t}\mathrm{e}}$	460	37.27 12.57
Required adsorbent mass (g)	$\mathrm{M}= {\uprho}_{\mathrm{b}} \mathrm{x} {\mathrm{V}}_{\mathrm{b}}$	10.65	211,950 ~ 211.95 kg

An initial nitrate removal efficiency of 67% was achieved using a fixed-bed column with a bed height of 10 cm and an internal diameter of 3 cm, operated at a flow rate of 5 mL/min. The equilibrium adsorption capacity of RDF500-2 at the bench scale was determined to be 18.29 mg/g. For scale-up, the Empty Bed Contact Time (EBCT) and equilibrium adsorption capacity were maintained constant. Since bulk density (ρ_b_ = 0.15 g/cm^3^) is a function of particle size distribution and shape, maintaining these parameters consistent ensures the minimum variations, still some deviations may occur in industries at the time of loading. The assumption is widely accepted for preliminary scale-up calculations and provides a basis for estimating adsorbent mass and bed dimensions. A bed length of 400 cm was selected based on the standard design criterion to ensure proper flow distribution and minimise channelling, with a corresponding bed volume (Vb = 1413 L), and the column diameter was 67.08 cm. Such heights are employed to ensure adequate contact time, efficient mass transfer, and optimal utilisation of the adsorbent bed. This configuration yielded an L/D ratio of 5–6, which is considered suitable for maintaining effective performance at high flow rates. The column diameter (67.08 cm) was derived from the bed volume relationship (V_b_ = πr^2^Z), where the required bed volume (1413 L) was calculated using the EBCT (V_b_ = Q × EBCT), and the bed height (Z = 400 cm) was selected based on design constraints (L/D = 6). Bulk density was determined under loose packing conditions following ASTM D1762-84 International to ensure the density and volume of the actual column operation. To achieve a targeted nitrate removal efficiency of 80%, the total packed adsorbent (RDF biochar- RDF500-2) within the column was estimated to be 211,950 g. Furthermore, the saturation time decreased significantly from 460 min at bench scale to 37.27 min in the scaled-up system, primarily due to the increased processing volume and optimised flow dynamics. The mass transfer zone calculations were carried out using equation- ${\mathrm{MTZ}}=Z{\hspace{0.17em}}(1-{t}_{b}/{t}_{s})$ with $Z=400\text{ cm}$ and the reported saturation time ${t}_{s}=37.27\text{ min}$ and ${t}_{b}={t}_{R}=12.57\text{ min}$, the MTZ length of 265 cm (approx. 66% of the packed bed). This large MTZ indicates under-utilisation of the bed (most of the column operates inside the transfer zone rather than as a sharp adsorption front), which explains the high adsorbent mass required.

The 80% nitrate removal was targeted based on the drinking water standards (WHO and EPA) and maximum permissible nitrate limit (40–45 mg/L as NO_3_^-^N). To achieve that standard, the experimental laboratory scale results needed to be about 70–80% of removal efficiency, with few published studies to support^86–88^. On comparing with literature studies carried out, granular activated carbon modified with ZnCl_2_ in a column study has shown a very low adsorption capacity of 1.76 mg/g^89^. In the comparative analysis (Table 7) of adsorption materials, RDF500-2 emerges as a promising alternative to conventional adsorbents, including activated carbon (AC), zeolite, and chitosan. Surface area, a critical parameter influencing adsorption capacity, is significantly lower in RDF biochar (10 m^2^/g) compared to AC, which typically ranges from 500 to 1500 m^2^/g due to its extensive activation treatment. Zeolite also exhibits a moderately high surface area (11.8–533.7 m^2^/g), whereas chitosan ranges from 100 to 500 m^2^/g.

**Table 7:** Comparative Performance of Adsorbent Materials for Scale-Up in Adsorption Column Design^90–92^.

Parameter	RDF Biochar	Activated Carbon (AC)	Zeolite	Chitosan-zeolite
Surface Area (m^2^/g)	10	901	8.691	213^93^
Bulk density (g/cm^3^)	0.15	0.5	1.2	0.6
Total pore volume (cm^3^/g)	0.009	0.490	0.026	-
Adsorption capacity (mg/g)	12–18	21.51	3.127	-
Column Efficiency (%)	65–75%	83%	-	87.23%
Cost per kg (USD/kg)	$1–2	$2–4	$0.30–0.50	$0.50–1.00
Regeneration feasibility	Limited	High	Moderate	Limited
Environmental impact	Low	High	Low	Low

Depending on its modification. This affects overall adsorption performance, as higher surface areas typically provide more active sites for pollutant uptake. Bulk density and total pore volume, which influence fixed-bed column performance, are significantly lower for RDF500-2 (0.15 g/cm^3^), making it advantageous for applications where a lighter adsorbent is preferred. The BET surface area value of 10 m^2^/g for RDF-derived char is significantly lower than that of conventional activated carbon (500–1500 m^2^/g); however, nitrate adsorption is primarily governed by surface functional groups and electrostatic interactions rather than surface area alone. The positively charged sites facilitate strong electrostatic attraction toward nitrate ions. The adsorption capacity of RDF500-2 (21.32 mg/g) was higher than that of AC (21.51 mg/g) and zeolite (3.127 mg/g). RDF-derived char possesses a heterogeneous pore structure with accessible active sites.

Furthermore, in fixed-bed column applications, RDF500-2 showed column efficiencies between 65–75%, which is closer to that of AC (83%), urging the optimisation in column design and pre-treatment strategies. From a cost perspective, RDF biochar has a significant advantage, with production costs estimated at $1-$2 per kilogram, especially when derived from municipal solid waste. This is considerably lower than AC ($2–4/kg) and far more economical than biopolymers like chitosan, which can cost over $10/kg due to their complex processing. Zeolite remains the cheapest among all ($0.3–0.5/kg), but it offers moderate adsorption and lower versatility in handling diverse pollutants. Lastly, in terms of environmental impact and regeneration potential, AC demonstrates high reusability, whereas biochar has a lower environmental impact and partial regeneration potential, making it more sustainable in the long term.

## Limitations of the study

The present study demonstrates the potential of RDF500-2 char as a sustainable and low-cost adsorbent for nitrate removal in a fixed-bed column. Several limitations are critically considered for real-world applications and future research. Firstly, the heterogeneity of the municipal solid waste (MSW)-derived RDF feedstock poses significant challenges for reproducibility and consistency in char quality. The RDF composition can vary significantly due to seasonal and regional variations in waste streams, leading to fluctuations in the physicochemical properties of the produced char, including surface area, porosity, and functional groups. Such variability could result in inconsistent adsorption performance during scale-up to industrial operations. Secondly, the adsorption capacity achieved in fixed-bed columns (21.32 mg/g) was substantially lower than that obtained under ideal batch conditions (160 mg/g). This discrepancy highlights the influence of non-equilibrium operating conditions, inadequate contact time, and mass transfer limitations, such as external film diffusion and intraparticle diffusion resistance, which were not fully resolved in this study. Moreover, the experiments were carried out using simulated nitrate solutions prepared from potassium nitrate (KNO₃), which do not replicate the complex matrix of real contaminated groundwater that often contains competing ions, such as sulfates, chlorides, and phosphates (future studies will focus on a multicomponent system). These coexisting ions can interfere with adsorption, significantly reducing nitrate removal efficiency. Another critical limitation concerns the chemical activation process using zinc chloride. While ZnCl_2_ modification enhances adsorption potential by increasing the number of active functional groups, it introduces additional environmental and operational concerns. ZnCl_2_ activation effectively enhances porosity, but the potential disposal of residual Zn species streams after activation, and the potential leaching into treated water have been partially explored^94^. Although proper post-treatment steps, such as thorough washing of RDF char with deionised water or acid-neutralisation, have effectively removed residual ZnCl_2_. Compared to other green activating agents (ex-, phosphoric acid, potassium hydroxide), ZnCl_2_ has been extensively studied and, when handled properly, does not pose greater environmental risks. The choice of ZnCl_2_ is often based on a balance between activation efficiency and safety. Sustainable end-of-life options, including regeneration of spent char, are also viable. Further, the fixed-bed column used in the study was limited to laboratory scale, with relatively low influent flow rates (3–7 mL/min) and small bed heights (5–10 cm). The transition from laboratory to industrial scale often encounters practical issues, such as hydraulic channelling, pressure drops, non-uniform flow distribution, and increased maintenance demands, which have not been experimentally validated. Additionally, the models employed to predict breakthrough curves are semi-empirical and assume ideal plug flow and homogeneous adsorption zones. Real systems often exhibit deviations due to axial dispersion, temperature gradients, and fouling, limiting the predictive accuracy of these models. The regeneration and reusability of the RDF char were not studied, leaving uncertainties regarding its operational lifespan and economic feasibility in continuous treatment systems. The cost advantage of RDF char could diminish over time due to the need for frequent replacement without regeneration. Furthermore, while the study focused exclusively on nitrate removal, groundwater contamination often involves a mixture of pollutants, including heavy metals, pesticides, and organic compounds. The performance of RDF char under multi-pollutant situations remains unexplored, limiting its generalizability. Life cycle assessment (LCA) and cost–benefit analyses are essential to establish its viability as a green technology.

## Conclusions

The study demonstrated that RDF500-2 is an effective, sustainable, and low-cost adsorbent for nitrate removal from aqueous solutions. The RDF500-2 achieved a maximum adsorption capacity of 21.32 mg/g, with a removal efficiency of 89% at optimised conditions of fixed-bed height (10 cm), flow rate (3 mL/min), and initial nitrate concentration (128 mg/L). Experimental results indicated that increasing the bed height enhanced adsorption by expanding the mass transfer zone, whereas higher flow rates reduced contact time. Among the five applied models, the Dose–Response model provided the most accurate prediction of breakthrough behaviour (R2 > 0.99), followed by the Thomas model. A scale-up design predicted that 211.95 kg of RDF char would be required to treat 100 L/min of nitrate-contaminated water with 80% efficiency, demonstrating the practical feasibility of the process. Comparative analysis revealed that chemically modified RDF char adsorption capacity is lower than that of commercial activated carbon. Its lower production cost ($1–2/kg) and minimal environmental impact make it ideal for large-scale implementation, particularly in developing regions. This study bridges solid waste valorisation with water purification, advancing the vision of “waste to treat waste” and contributing to circular economy principles in sustainable environmental management.

## Supplementary Information

Supplementary Information.

## Data Availability

All data supporting the findings of this study are available within the paper and its Supplementary Information.
