# Supplementary material for: Sustainable char production from pyrolysis of refuse-derived fuel for nitrate removal from water
Source: Sci Rep. 2026 May 21;16:27859. doi: 10.1038/s41598-026-51297-1 (PMC13547355; doi:10.1038/s41598-026-51297-1)
Supplement: Supplementary file 1 [file 41598_2026_51297_MOESM1_ESM.docx]

**Sustainable production of char from pyrolysis of refuse-derived fuel and its application for nitrate removal from water: Adsorbent Generation and Column Optimisation**

Yashasvi Trivedi ^a^, Abhishek Sharma^a, d*^, Manisha Sharma^a^, Ranjeet Kumar Mishra^b*^, Jyeshtharaj B Joshi^c^

^a^Department of Biotechnology and Chemical Engineering, Manipal University Jaipur, Rajasthan, 303007, India

^b^ Manipal Institute of Technology, Manipal Academy of Higher Education, Manipal, India.

^c^ Department of Chemical Engineering, Institute of Chemical Technology, Mumbai-400019, Maharashtra, India.

^d^ Department of Chemical Engineering, BITS Pilani K. K. Birla Goa Campus, Zuarinagar, Sancoale, Goa, 403726, India.

Corresponding Author's details:

[abhishek.sharma@jaipur.manipal.edu](mailto:abhishek.sharma@jaipur.manipal.edu); (Abhishek Sharma), [ranjeet.mishra@manipal.edu](mailto:ranjeet.mishra@manipal.edu) (Ranjeet Kumar Mishra)

**Fig. S1**. RDF char preparation

**Fig. S2**. Experimental fixed bed column setup

 **Fig. S3**. Breakthrough curve for nitrate adsorption onto RDF500-2 at different a) bed height (5, 7.5 and 10 cm, b) Flow rate (3, 5 and 7 mL/min) and c) initial adsorbate concentration (67 mg/L, 128 mg/L and 185 mg/L) pH=6.8: Wolborska model fitting

The fixed-bed column system was designed and scaled up from the laboratory to the industrial scale using RDF char as the adsorbent, targeting an 80% removal efficiency. The scale-up was based on a flow rate of 100 L/min, corresponding to an approximate 20,000-fold increase from the laboratory setup. Fundamental equations for column design and scale up have been provided below.

Step 1. Bed volume

$$Bed volume (V_{b})=Q x EBCT$$

where Q is flow rate in L/min and EBCT is the empty bed contact time (min) or the empty bed residence time (min) [68]. The bed volume was calculated using the EBCT relationship (V_b_ = Q × EBCT), followed by column dimensions derived from V_b_ = πr²Z with defined assumptions for bed height and L/D ratio.

$${\mathbf{Required} \mathbf{bed} \mathbf{volume}, \mathbf{V}}_{\mathbf{b}}=100 x 14.13= 1413 L=1413000 cm^{3}$$

Step 2. Length of the bed

$$Bed Volume, V_{b}=\pi r^{2}Z$$

Assuming,

Bed length (Z) = 400 cm

$$r^{2}=\frac{1413000}{1256}=1125$$

Required Diameter of the Column, D = 67.08 cm

$$\frac{\mathbf{L}}{\mathbf{D}}=\frac{400}{67.08}=5.96 \approx\mathbf{6}$$

Where L=Z is the length of the adsorbent bed (cm) and D is the diameter of the column (cm).

Step 3. Porosity of the RDF char (adsorbent)

The biochar particle size (2.5 mm) corresponds to a granular material typically used in fixed-bed columns.

$${\mathbf{Bulk} \mathbf{density}, \boldsymbol{\rho}}_{\mathbf{b}}=\frac{\mathrm{Mass}}{\mathrm{Volume}}=\frac{10.65}{70.65}=0.15 g/cm^{3}$$

$$\mathbf{Particle} \mathbf{density}, \boldsymbol{\rho}_{\mathbf{p}}=\frac{Total Mass of sample}{Volume displaced}=\frac{10.65}{8.02}=\mathbf{1}.\mathbf{32} g/cm^{3}$$

$\mathbf{Porsoity}, \boldsymbol{\varepsilon}=1 - \frac{\boldsymbol{\rho}_{\mathbf{b}}}{\boldsymbol{\rho}_{\mathbf{p}}}=1- \frac{Bulk density}{Particle density}= 1-\frac{0.15}{1.32}=\boldsymbol{0}.\boldsymbol{89}$

Bulk density remains constant during scale-up with similar packing conditions and particle size (1.5 mm in both systems). The porosity was determined from independently measured bulk and particle densities (ε = 1 − ρ_b_/ρ_p_), ensuring consistency with packing characteristics.

Step 4. Adsorbent Mass Required

$\mathbf{M}\left( \mathbf{g} \right)=\rho_{b} x V_{b}=Bulk density x Required bed volume=0.15 x 1413000=211950 g\approx211.95 kg$

$$Equilibrium nitrate adsorption capacity, \boldsymbol{q}_{\boldsymbol{e}}=\frac{m_{ad,t_{s}}}{M}=18.29 mg/g= \frac{m_{ad,t_{s}}}{211950}$$

$${\mathbf{Amount} \mathbf{of} \mathbf{nitrate} \mathbf{adsorbed}, \mathbf{m}}_{\mathbf{ad},\mathbf{t}_{\mathbf{s}}}=3876565.5 \mathrm{mg}$$

The total adsorbent mass was then calculated using M=ρ_b_ × V_b_, and adsorption capacity was used to estimate nitrate uptake and saturation time based on mass balance relationships.

Now, to achieve 80% of the nitrate removal efficiency

$$\boldsymbol{Y}=\frac{m_{ad,t_{s}}}{m_{in,t_{s}}}=0.80 \left( \mathrm{given} \right)=\frac{3876565.5}{C_{o}Qt_{s}}= \frac{3876565.5}{1300 x 100 x t_{s}}$$

${\mathbf{Saturation} \mathbf{time},\mathbf{t}}_{\mathbf{s}}=37.27 min$

$$C_{o}Qt_{s}=\mathbf{m}_{\mathbf{in},\mathbf{t}_{\mathbf{s}}}= 4845706.87 mg$$

Finally, the effective residence time was computed using RT = (V_b_ × ε)/Q to account for void fraction within the bed.

Step 5. Effective Residence time

$$Residence time (min)=\frac{V_{b} x \varepsilon}{Q}= \frac{Bed volume x porosity of\mathrm{bed}}{Flow rate}$$

$$\mathbf{RT} \left( \mathbf{min} \right)=\frac{1413000 x 0.89}{{10}^{4}}=12.57 min$$
